# Mechanisms of breast cancer treatment using *Gentiana robusta*: evidence from comprehensive bioinformatics investigation

**DOI:** 10.1038/s41598-024-76063-z

**Published:** 2024-12-30

**Authors:** Bo Xiong, Xinxin Zhang, Dongzhi Sangji, Lianghong Ni, Mingjie Fan, Beibei Fan

**Affiliations:** 1 Department of Clinical Pharmacy, Baoshan Hospital Affiliated to, Shanghai University of Traditional Chinese Medicine, Shanghai, China; 2 Shanghai University of Traditional Chinese Medicine, Shanghai, China; 3 Tibetan Medical Hospital of Xizang Autonomous Region, Lhasa, China; 4 School of Pharmacy, Shanghai University of Traditional Chinese Medicine, Shanghai, China; 5 Department of Pharmacy, Shanghai Fourth Rehabilitation Hospital, Shanghai, China

**Keywords:** *Gentiana robusta*, Breast cancer, Network pharmacology, Molecular docking, Molecular dynamics simulation, Biochemistry, Computational biology and bioinformatics, Molecular medicine

## Abstract

This study investigates the potential treatment of breast cancer utilizing *Gentiana robusta* King ex Hook. f. (QJ) through an integrated approach involving network pharmacology, molecular docking, and molecular dynamics simulation. Building upon prior research on QJ’s chemical constituents, we conducted Gene Ontology (GO) and Kyoto Encyclopedia of Genes and Genomes (KEGG) pathway analysis using the DAVID database. Network interactions and core genes were identified using Cytoscape 3.9.1. Key target genes, including Interleukin-6 (IL-6), tumour suppressor gene P53 (TP53), and epidermal growth factor receptor (EGFR), were selected for molecular docking with QJ’s active components, 2′-O-β-D-glucopyranosyl-gentiopicroside and macrophylloside D, employing Schrodinger Maestro 13.5. Molecular dynamics (MD) simulations were performed using the Desmond program. A total of 270 intersection targets of active ingredients and diseases were identified, with three core targets determined through network topology screening. Enrichment analysis highlighted the involvement of QJ in breast cancer treatment, primarily through the hsa05200 cancer signaling pathway and the hsa04066 HIF-1 signaling pathway. Molecular docking and dynamics simulations demonstrated the close interaction of 2′-O-β-D-glucopyranosyl-gentiopicroside (QJ17) and macrophylloside D (QJ25) with IL6, TP53, and EGFR, and other target genes, showcasing a stabilizing effect. In conclusion, this study unveils the effective components and potential mechanisms of 2′-O-β-D-glucopyranosyl-gentiopicroside and macrophylloside D in breast cancer prevention and treatment. The identified components act on target genes such as IL6, TP53, and EGFR, regulating crucial pathways including the cancer signaling and Hypoxia-inducible factor 1 (HIF-1) signaling pathways. These findings provide valuable insights into the therapeutic potential of QJ in breast cancer management. However, further experimental research are needed to validate the computational findings of QJ.

## Introduction

Breast cancer has emerged as the predominant female malignancy, surpassing lung cancer to claim the title of the most common cancer globally^1^. The escalating incidence, attributed to heightened exposure to risk factors, underscores the urgent need for effective treatments. In the realm of clinical intervention, individualized approaches such as surgery, radiotherapy, chemotherapy, endocrine therapy, and molecular targeted therapy are the primary means of treatment.

Despite the prevalent use of various drug therapies, Western medicines employed in breast cancer treatment often come with severe adverse reactions, including liver and kidney toxicity, gastrointestinal issues, allergic reactions, and bone marrow suppression. Recognizing these challenges, the medical community is turning its attention towards alternative approaches, particularly Traditional Chinese medicine (TCM) and other natural drugs. These modalities offer notable advantages, including high efficacy, fewer side effects, and the ability to target multiple pathways.

The promising prospects of TCM and natural drugs in anti-tumor treatments have positioned them as focal points in the ongoing research and development of anticancer drugs^2,3^. This shift towards exploring diverse treatment modalities reflects the growing recognition of the need for holistic and personalized approaches to tackle the complex challenges posed by breast cancer.

China’s abundance in natural medicinal resources is complemented by the diversity of its traditional ethnic medicines, with Tibetan medicine standing as a significant component of Chinese traditional medicine. Our research group has undertaken multiple expeditions to the Qinghai-Tibet Plateau throughout the year, aiming to collect scientific research samples and explore the rich tapestry of Tibetan medicinal resources.

Amidst the myriad of medicinal plants, QJ emerged as a focal point of our attention. This perennial herb, belonging to Sect. *Cruciata Gaudin *of *Gentiana *genus, thrives in the southern Tibetan region. Revered as one of the original plant sources in “Jieji” Tibetan medicine, QJ boasts a storied history of medicinal usage. Known for its therapeutic effects in dispelling wind and dampness, clearing heat and the gallbladder, soothing tendons, and relieving pain, it has found extensive clinical application in treating conditions such as rheumatoid arthritis, low-fever night sweats, jaundice hepatitis, various forms of bleeding, swelling, and other "Chi-ba" diseases^4–6^.

Notably, QJ serves as a primary raw material for several proprietary Chinese medicines, including Shisanwei Bang-Ga powder, Ershiwuwei Datang pills, and more^7^. This underscores its significance not only in traditional Tibetan medicine but also in the broader landscape of Chinese herbal formulations.

In essence, the exploration of QJ and Tibetan medicine not only contributes to our understanding of traditional healing practices but also holds promise for the development of novel therapeutic interventions. The unique ecological context of the Qinghai-Tibet Plateau adds an extra layer of richness to the potential discoveries in the realm of medicinal plants and their applications.

To date, research on QJ has primarily centered on the identification of raw materials, with limited attention given to specific components such as gentiopicroside, loganic acid, swertiamarin, other iridoid terpenoids, roburic acid, and stigmasterol^8^. In order to delve deeper into the contemporary pharmacological actions and clinical applications of QJ, we employed ultra-high-performance liquid chromatography-electrospray ionization with quadrupole time-of-flight tandem mass spectrometry (UHPLC-ESI-Q-TOF–MS/MS) technology for a comprehensive analysis of its chemical composition^9^.

The association between inflammation and tumors is well-established, with inflammation recognized as the eighth biological feature in malignant tumors, influencing their occurrence, development, invasion, and metastasis^10,11^. In this context, QJ’s role in managing inflammatory conditions becomes particularly relevant in the broader framework of cancer research.

Natural medicine is inherently complex, featuring numerous components, targets, and action pathways. Network pharmacology has proven instrumental in understanding the relationship between TCM and modern pharmacology, providing insights into the overall mechanisms of action for TCM compounds and facilitating the analysis of drug compatibility laws and formulations. This innovative approach sparks new ideas for studying intricate TCM systems^12,13^.

Molecular docking technology, a simulation method predicting the interaction between small molecule ligands and receptors, along with MD simulation, addressing dynamic molecular behavior, are integral tools in drug design. The combination of these approaches enhances the precision and efficiency of drug design, contributing to the discovery of novel drugs and elucidating the mechanisms underlying drug treatments^14,15^.

In this study, we aimed to unravel the potential breast cancer-resistant mechanism of QJ. Employing state-of-the-art UHPLC-ESI-Q-TOF–MS/MS detection, we meticulously determined the chemical composition of QJ. Additionally, we harnessed the power of network pharmacology, molecular docking, and MD simulation, utilizing diverse biological information analysis methods^16^.

To identify the active components linked to breast cancer target genes, an exhaustive screening process was conducted on QJ. Subsequently, molecular docking and MD simulation were employed to predict the binding sites of small molecule active components with target genes.

## Methods and materials

### Target prediction and validation

**Fig. 1:**
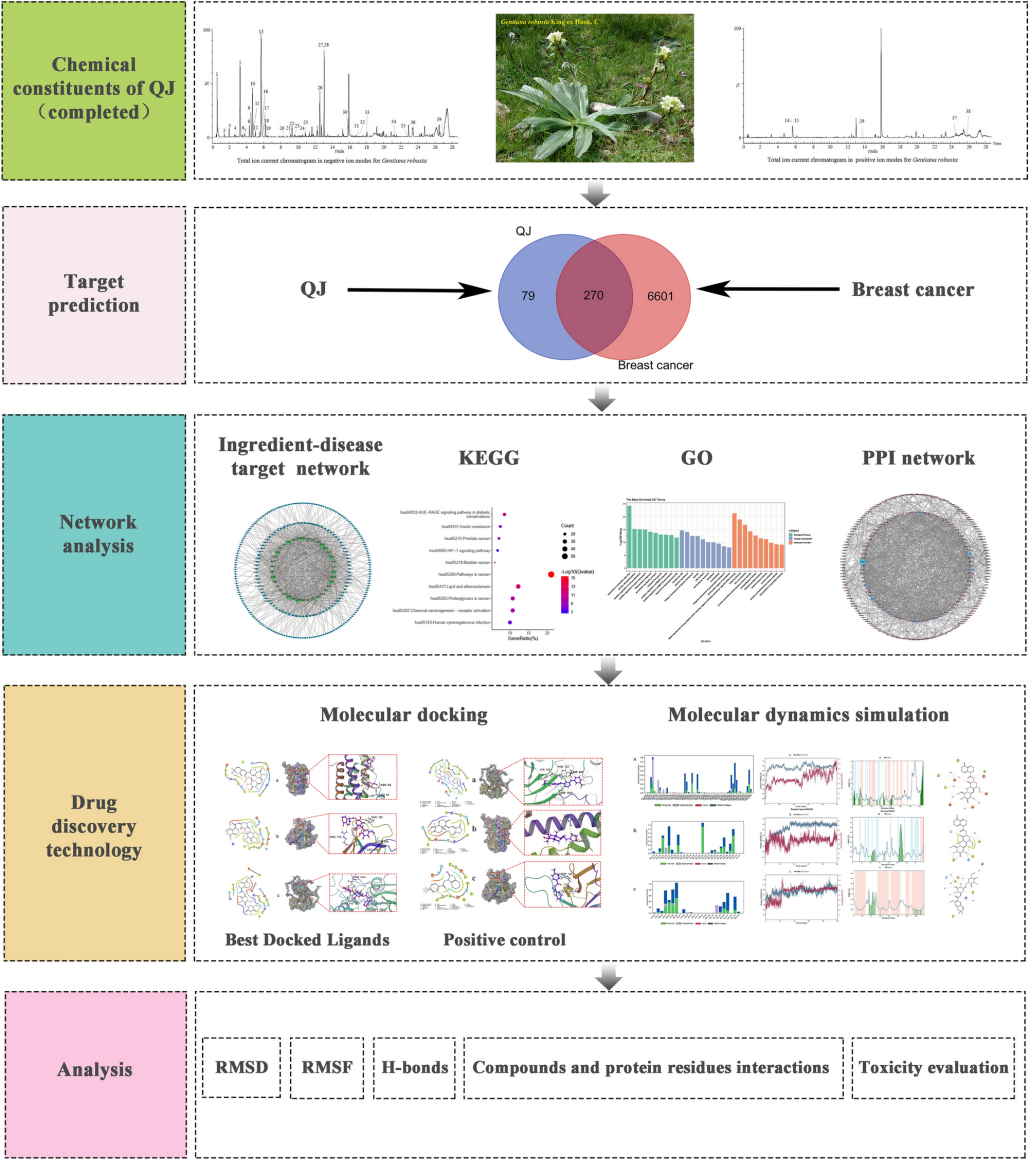
Flowchart of this study.

The flowchart showing the outline of this study is presented in Fig. 1. Based on our previous studies on the chemical composition analysis of QJ, all 39 compounds in QJ(Table S1) were selected for the prediction of biological targets. Using the PubChem database, (https://pubchem.ncbi.nlm.nih.gov/) information regarding the 39 active ingredients was retrieved. The 2D SDF file was input to the Swiss Target Prediction platform^17^, with an aim-listed probability threshold of 0.1 or higher. Additionally, the active ingredient target was screened. The MalaCards (https://www.malacards.org/), OMIM (https://omim.org/), and DisGeNET (https://www.disgenet.org/) databases were utilized^18^. Furthermore, to obtain the disease target genes, “breast cancer” was applied as the search term and the species was set to human.

## Drug-Component-Target-Disease Network Construction

The targets of the screened active ingredients were intersected with the targets of breast cancer and imported into Venny 2.1 software. They were then displayed on a Venn diagram and used as potential targets of drug action for subsequent analysis. To better understand the complex relationship between the active ingredients of TCM and the corresponding disease targets, a composition-target-disease network map was constructed using Cytoscape 3.9.1 software. This network map was based on the QJ active ingredients, active ingredient targets, and breast cancer disease targets.

## GO and KEGG pathway enrichment analysis

The QJ active ingredients and common breast cancer disease targets were imported into DAVID Bioinformatics Resources 6.8 (https://david.ncifcrf.gov/home.jsp) for KEGG and GO pathway enrichment analysis. With P < 0.05 as the threshold, the top ten enrichment items were displayed in both a column chart and a bubble chart. The KEGG database was used to map the proteins associated with the relevant signaling pathways to the KEGG pathway.

## Protein–protein interaction(PPI) network construction and key target screening

The common drug disease targets were entered into the STRING v12.0 database (https://string-db.org/cgi/input.pl). To construct the PPI network^19^, the biological species was set as “*Homo sapiens*” and the threshold was set as the default parameter. The TSV file obtained from the STRING database was imported into Cytoscape 3.9.1 software^20^. Next, a topological analysis of the PPI network was performed using three centrality algorithms, including degree centrality, intermediate centrality, and proximity centrality.

## Molecular docking

A molecular docking study was performed to predicted the binding situation of protein and small molecules. The crystal structures were obtained from the RCSB PDB database (https://www.rcsb.org/) based on the principle of species as human source, release time as new as possible and high precision. Following that, the Protein Preparation Wizard module of Schrodinger Maestro 13.5 was used for pretreatment. The SiteMap module and Receptor Grid Generation module in Schrodinger were then used to obtain the active site of the protein. Next, the 2D SDF structure file corresponding to the active ingredients was downloaded from the PubChem database and pre-processed using the LigPrep module in Schrodinger. Finally, all the treated ligand compounds were linked to the active sites of the top three proteins for XP molecular docking and MM-GBSA analysis. The binding sites were visualized using Schrodinger software^21^.

Meanwhile, the top three target proteins and their corresponding positive drugs were selected for molecular docking according to the prediction results of key targets screening.

## MD simulation

To further optimize the binding mode of the protein-peptide complexes, the compounds with the best binding interactions and free energies over the target proteins were chosen to performe conventional MD simulations using the Desmond v5.9 Schrödinger program. The orthogonal partial least squares-2005(OPLS-2005)^22^ force field was employed to parameterize the protein and small molecules, while the TIP3P model was used for the water solvent. The protein-small molecule complex was placed in a cubic water box and solvated. Next, the system charge was neutralized by adding 0.150 M chloride and sodium ions. The energy of the system was initially minimized using the steepest descent minimization method for 50,000 steps. Subsequently, the positions of the heavy atoms were restrained for NVT and NPT equilibration for an additional 50,000 steps. The system temperature was maintained at 300 K and the system pressure was kept at 1 bar. After completing the two equilibration stages, an unrestricted simulation was performed for 100 ns. The interactions were analyzed and dynamic trajectory animations were generated using Maestro 2023.

## Results

### Active ingredient target prediction, venn diagram and PPI network construction

**Fig. 2:**
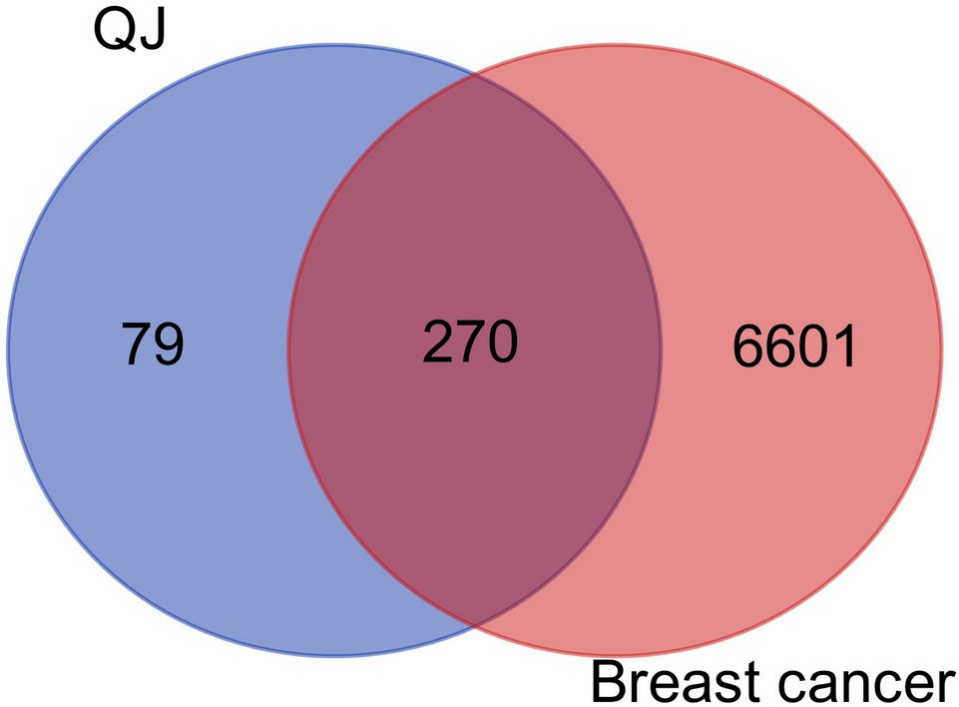
Venn diagram of potential QJ targets for the treatment of breast cancer.

**Fig. 3:**
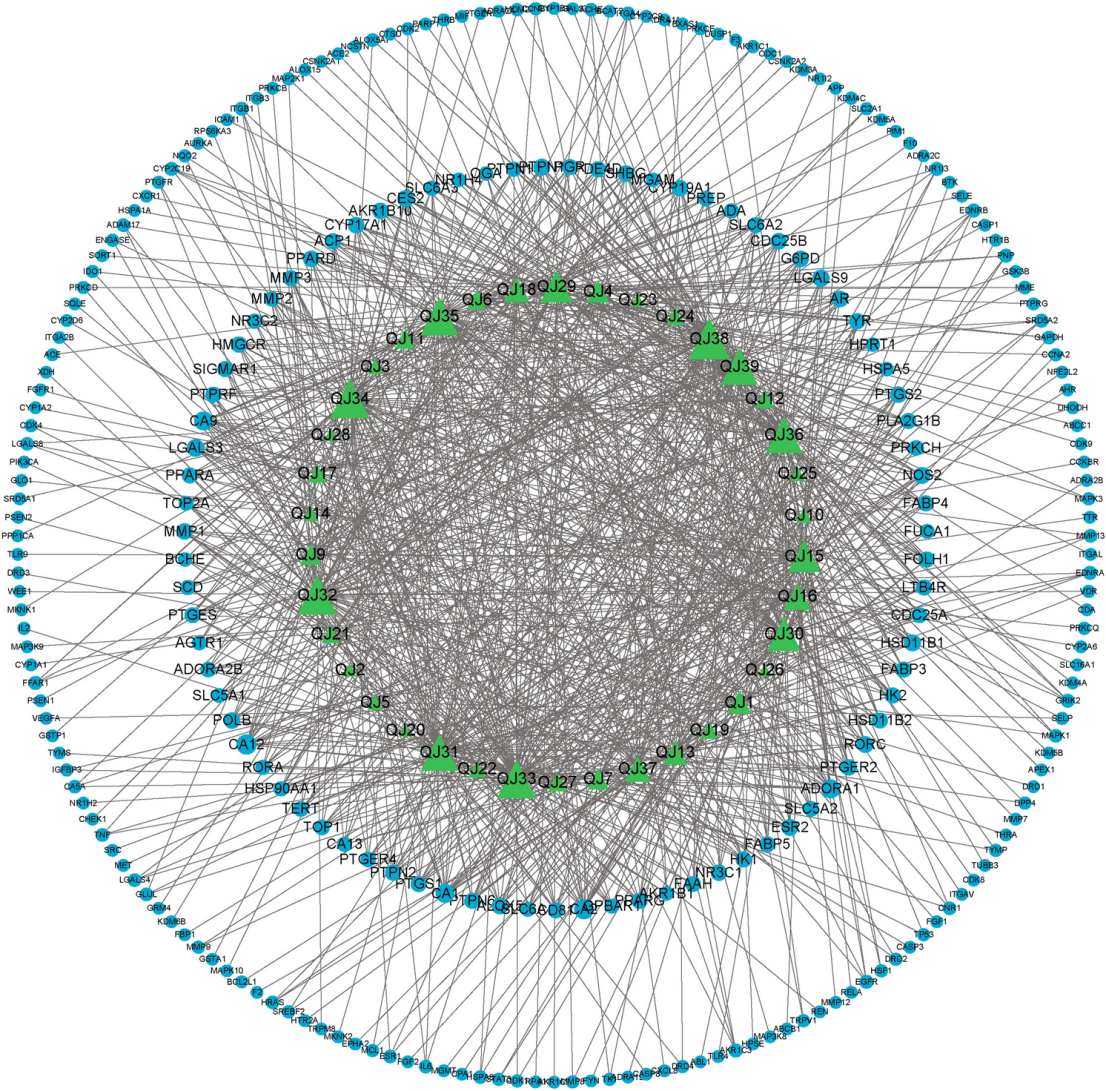
QJ active ingredient-breast cancer disease target network. Images were created using Cytoscape 3.9.1 software.

A total of 349 active ingredient targets screened and 6,871 demerged disease targets were analyzed to display how they intersected. Ultimately, 270 common targets were identified as potential drug action targets for subsequent analysis (Fig. 2). To better understand the complex interaction between the active ingredients of TCM and their corresponding disease targets, a composition-target-disease network diagram was constructed based on the QJ active ingredients, active ingredient targets, and breast cancer disease targets (Fig. 3).

## GO and KEGG enrichment analysis

**Fig. 4:**
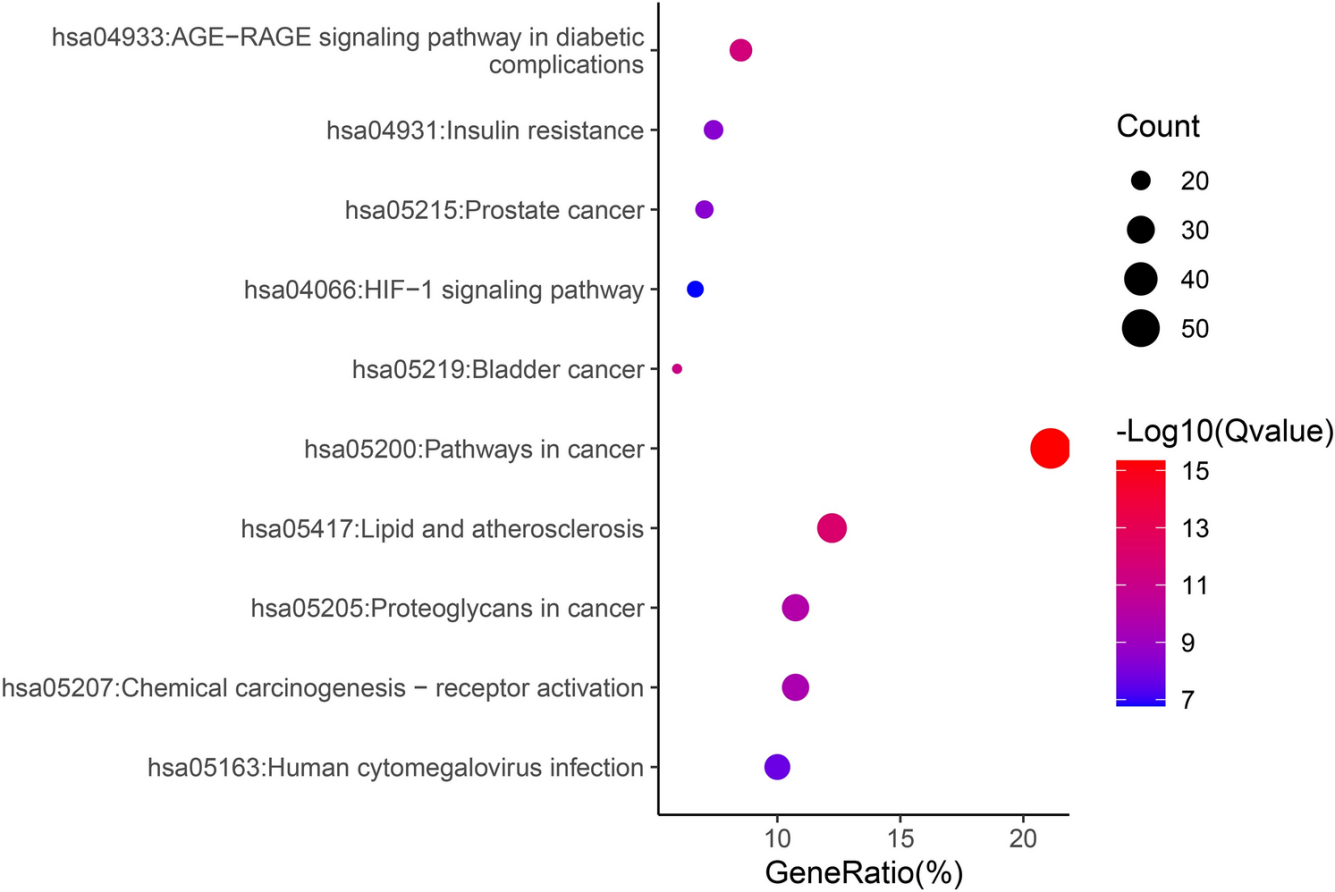
KEGG enrichment analysis bubble diagram.

**Fig. 5:**
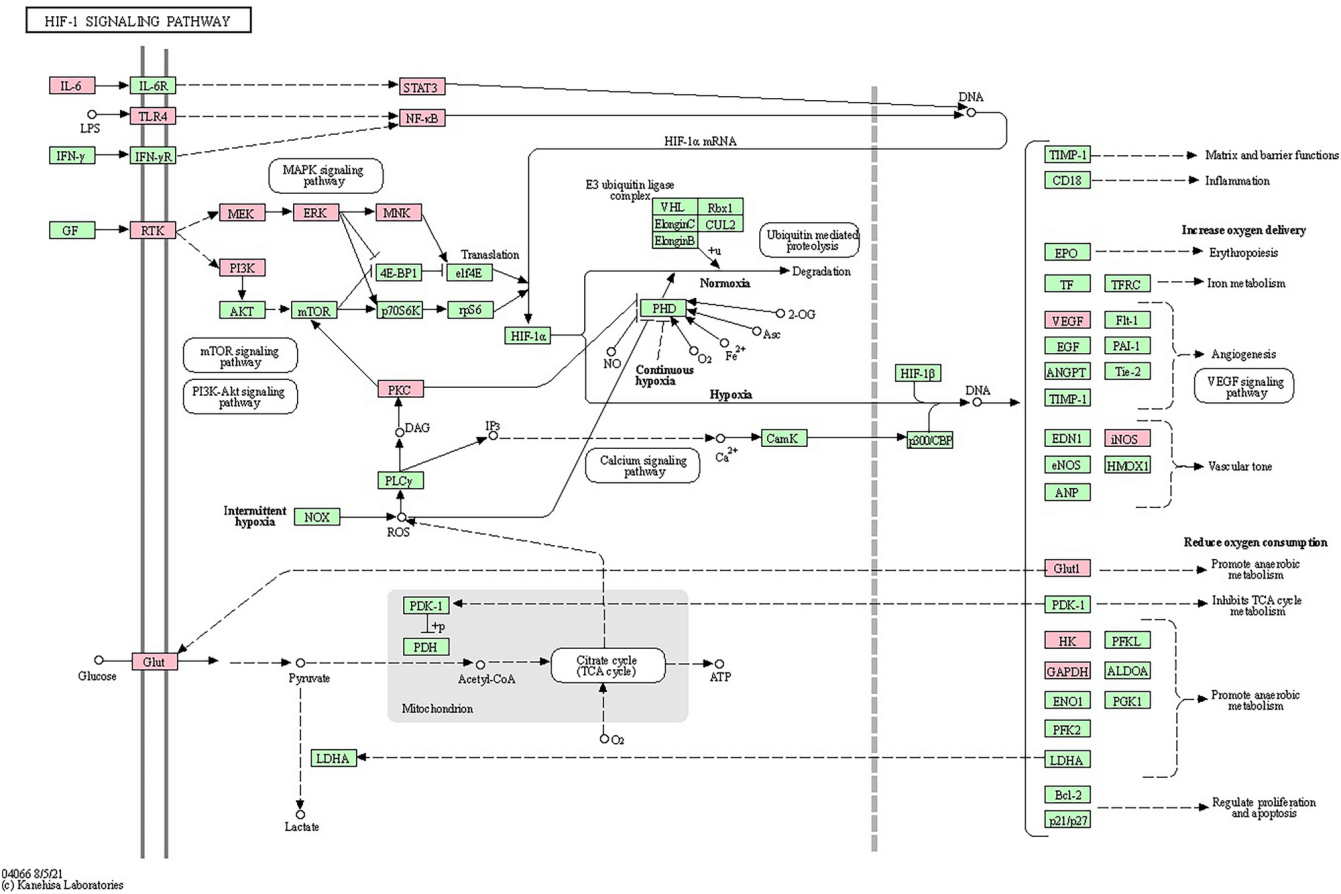
Related target action relationship between QJ and the HIF-1 signaling pathway. Red rectangles represent key targets for QJ intervention. Diagrams created with KEGG. (Copyright Permission 241176).

**Fig. 6:**
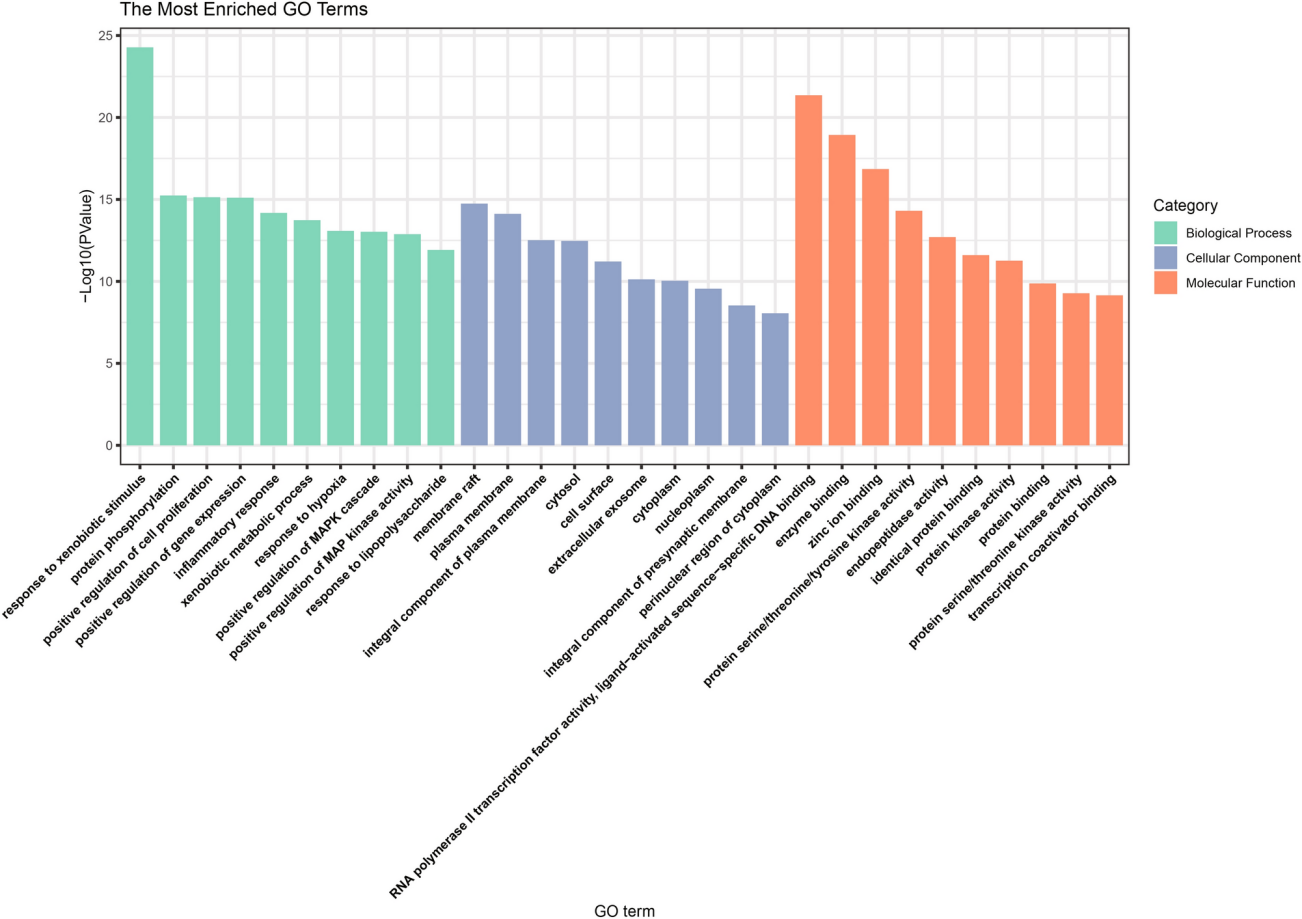
Top ten significantly enriched terms in BP, CC, and MF of the GO analysis.

GO analysis is used to describe the biological functions of targets which include biological processes (BP), molecular functions (MF), and cellular components (CC). The KEGG is a database which has the ability to perform functional enrichment analysis power. We proceeded to analyze the 270 commonly owned targets for KEGG pathway^23–25^ and GO enrichment assays. The findings indicated that among these targets, the most enriched pathway was "hsa05200: cancer signaling pathway," which is highly pertinent to our focus on cancer treatment (Fig. 4). Subsequently, we directed our attention to the "hsa04066: HIF-1 signaling pathway," given its close association with breast cancer, the subject of our current study. Figure 5 illustrates the significant impact of QJ active ingredients on each major node of this HIF-1 pathway. Furthermore, the GO enrichment analysis unveiled that the primary enriched biological process was "response to external stimuli," while the main enriched cellular components were membrane rafts (Fig. 6). Additionally, the primary enriched molecular functions identified were "RNA polymerase II transcription factor activity" and "ligand activation sequence-specific DNA binding."

## PPI network construction and key target screening

**Fig. 7:**
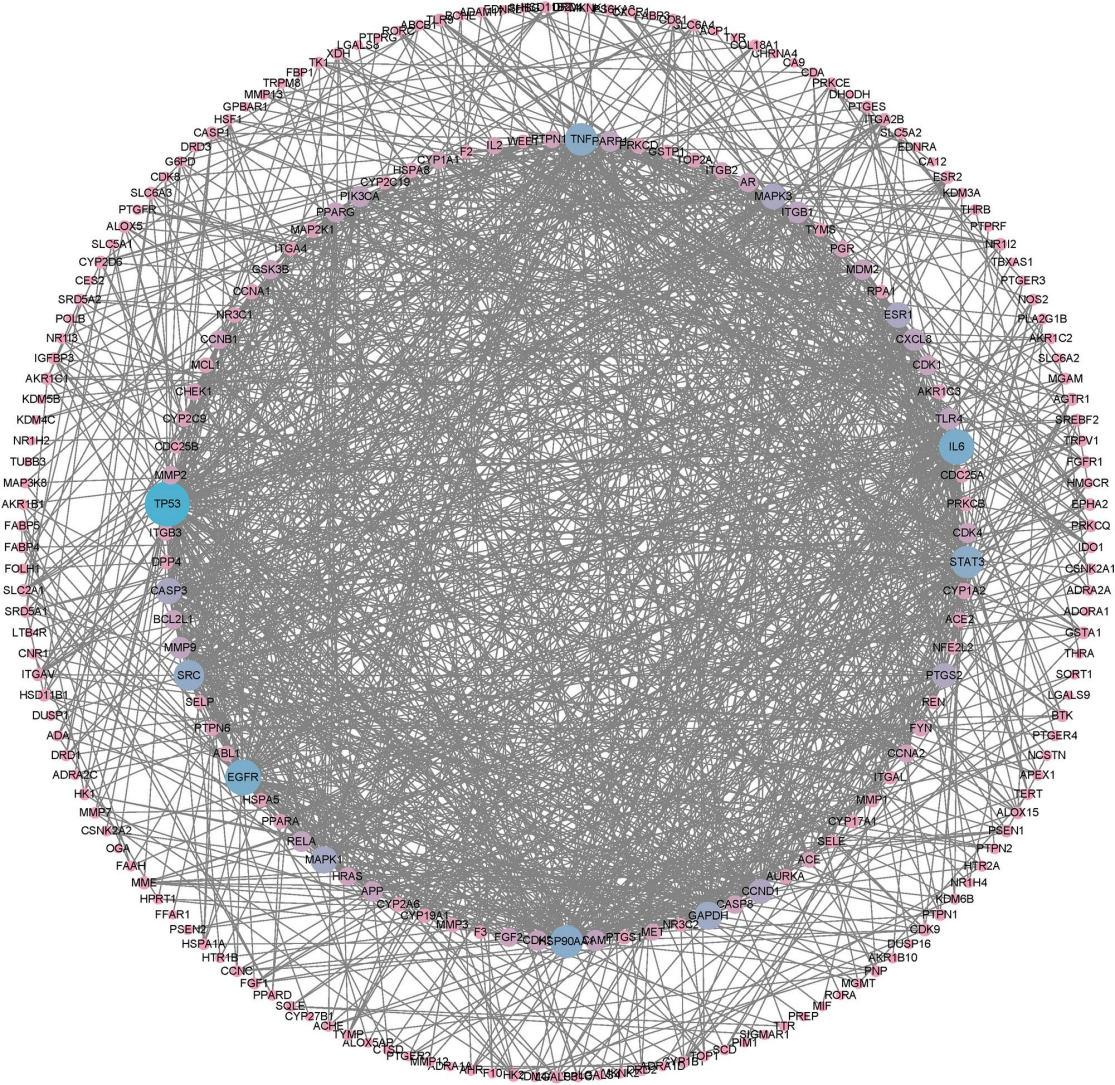
PPI network diagram of the intersection targets. Images were created using Cytoscape 3.9.1 software.

The common disease targets of drugs were entered into the STRING database to construct the PPI network (Fig. 7). Next, the TSV file obtained from the STRING database was further analyzed for key targets of the PPI network the results are listed(Table S2). The top three key targets in the three centrality algorithms are TP53, EGFR and IL6 genes (Table 1).

**Table 1:** Top ten topological analysis of the PPI network based on three centrality algorithms.

Target name	Degree centrality	Target name	Betweenness centrality	Target name	Closeness centrality
TP53	75	TP53	0.185069	TP53	0.520343
EGFR	56	IL6	0.088949	EGFR	0.492901
IL6	55	EGFR	0.0818	IL6	0.486
HSP90AA1	50	SRC	0.071918	SRC	0.483101
TNF	49	PTGS2	0.0643	GAPDH	0.481188
STAT3	48	TNF	0.060704	STAT3	0.478346
SRC	45	HSP90AA1	0.056621	HSP90AA1	0.472763
GAPDH	40	ESR1	0.053633	TNF	0.47093
MAPK1	38	GAPDH	0.051498	MAPK1	0.461977
CASP3	36	STAT3	0.046388	ESR1	0.453358

## Validation by molecular docking

Molecular docking simulations showed that QJ17 and QJ25 which are the major active compounds in QJ performed better than other compounds against the EGFR, IL6 and TP53 target proteins (Table S3, S4, S5). The QJ17 and QJ25 compounds were subjected to molecular docking to confirm their binding to EGFR, p53, and IL-6 proteins. Utilizing the XP mode, a sophisticated computational approach tailored for higher-resolution molecular docking on specific targets, we employed flexible docking, allowing both the protein and the ligand to adjust. The subsequent MM-GBSA analysis corroborated the findings from the XP docking. The results from these analyses were denoted as XP Gscore and MM-GBSA dG Bind, respectively. The XP Gscore serves as a docking score, reflecting the binding free energy, while the MM-GBSA dG Bind quantifies the binding strength of the ligand and the protein. A stable binding between the ligand and the protein is indicated when the XP Gscore is less than -6. Moreover, an MM-GBSA dG Bind value lower than -30 kcal/mol signifies low binding free energy, indicative of stable binding between the ligand and the protein.

**Fig. 8:**
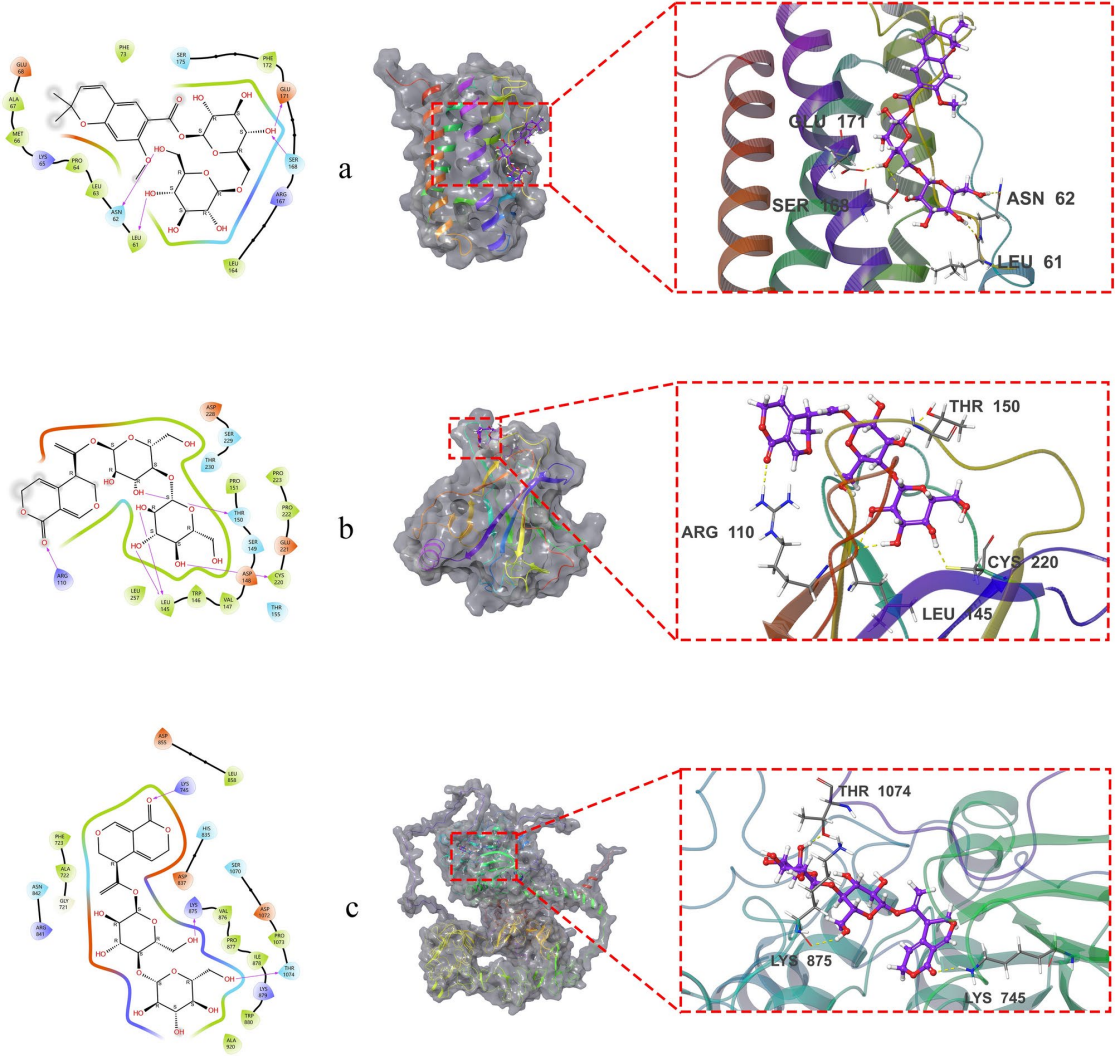
Two and three-dimensional images of compounds QJ17 and QJ25 docking with EGFR, TP53, and IL6 protein: (**a**) QJ25 docking with IL6; (**b**) QJ17 docking with TP53; (**c**) QJ17 docking with EGFR. Hydrogen bonds are represented by yellow dotted lines.

A thorough examination of the XP docking results and the MM-GBSA analysis unveiled that QJ25 exhibited the most favorable docking performance with the IL-6 protein, boasting an XP Gscore of -8.528 and an MM-GBSA dG Bind of -33.17 kcal/mol (Table 2). Both the docking score and free binding energy were notably low, indicating a stable interaction between QJ25 and the IL-6 protein. Following closely, p53 and QJ17 displayed the second-best performance, with an XP Gscore of -8.283 and an MM-GBSA dG Bind value of -31.32 kcal/mol, respectively. Furthermore, the MM-GBSA dG Bind values of EGFR and QJ17 surpassed -30 kcal/mol, suggesting relatively stable binding between QJ17 and both EGFR and p53 proteins. In essence, both QJ17 and QJ25 compounds exhibited stable binding with IL-6, p53, and EGFR proteins, underscoring their potential anti-breast cancer activity by modulating the expression of these proteins to some extent. Figure 8 illustrates that both compounds were bound to the active pocket surface of IL-6, p53, or EGFR proteins.

**Table 2:**
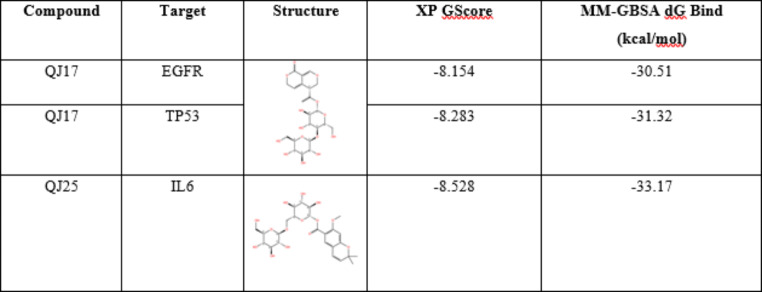
XP and MM-GBSA results.

The QJ25 compound engaged in hydrophobic interactions with specific residues of the IL-6 protein, namely GLU171, SER168, ASN62, and LEU61. Conversely, QJ17 established hydrophobic interactions with residues of the p53 protein, including THR150, CYS220, ARG110, and others. Furthermore, QJ17 formed two hydrogen bonds with the p53 protein residue LEU145, while also interacting via hydrogen bonding with residues LYS745, LYS875, and THR1074. In the docking study, it was observed that QJ17 obstructed the active site residues of EGFR proteins, including LYS:745, LYS:875, and THR:1074, through intermolecular hydrogen bonding. Additionally, QJ17 impeded the active site residues of p53 proteins, such as ARG:110, LEU:145, THR:150, and CYS:220, through intermolecular hydrogen bonding. Similarly, QJ25 hindered the active site residues of IL-6 proteins, namely ASN:62, LEU:61, GLU:171, and SER:168, through intermolecular hydrogen bonding.

**Fig. 9:**
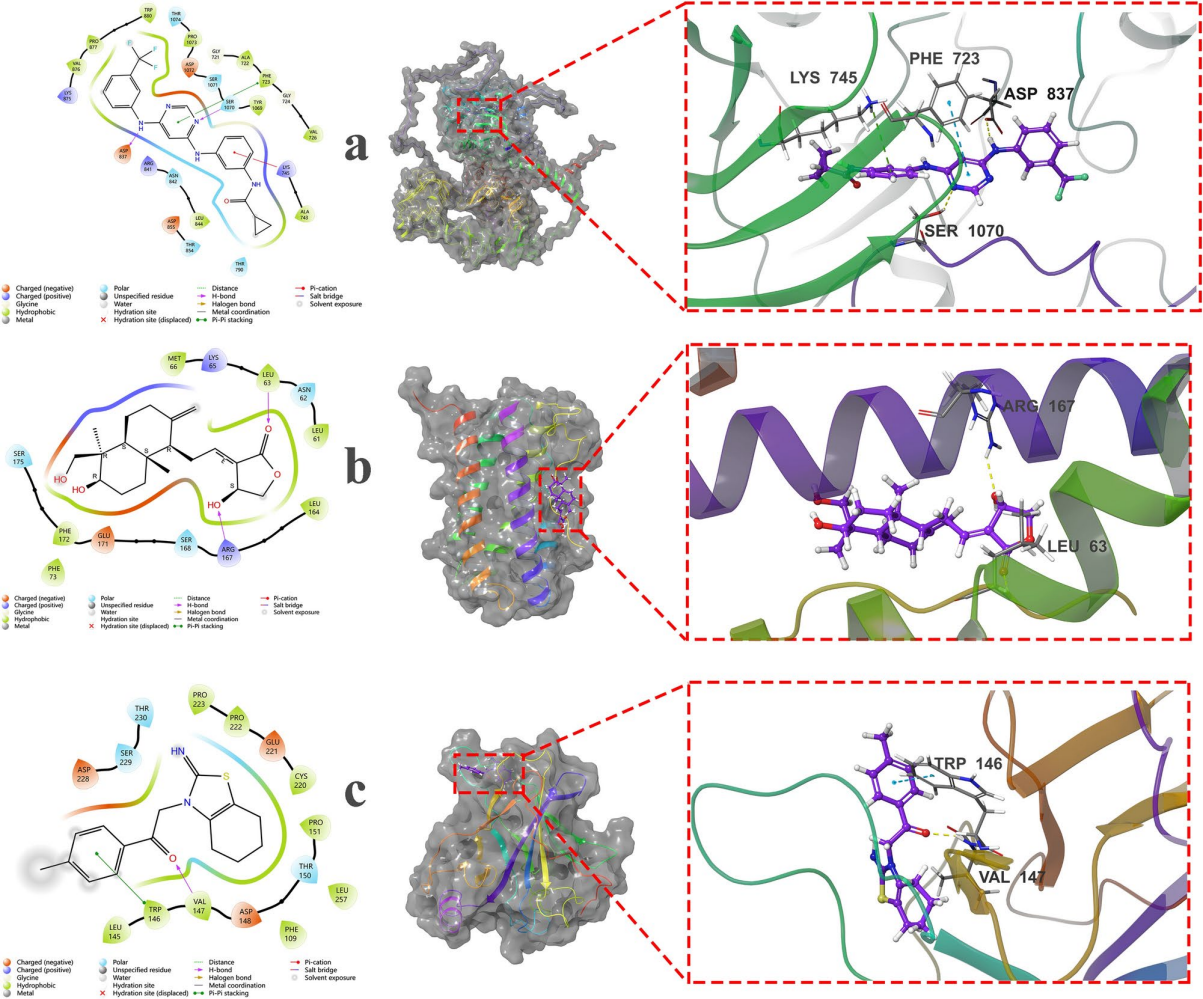
Two and three-dimensional images of compounds EGFR Inhibitor, Andrographolide and Pifithrin-α hydrobromide docking with EGFR, IL6 and TP53 protein: (**a**) EGFR Inhibitor docking with EGFR; (**b**) Andrographolide docking with IL6; (**c**) Pifithrin-α hydrobromide docking with TP53. Hydrogen bonds are represented by yellow dotted lines, π-π bonds are represented by blue dotted lines and π-Cation bonds are represented by green dotted lines.

Positive control inhibitor for EGFR, IL6 and TP53: EGFR Inhibitor^26^, Andrographolide^27^ and Pifithrin-α hydrobromide^28^ were selected respectively. The positive drug exhibited stable binding with IL-6, TP53, and EGFR proteins (Table 3). Figure 9 illustrates that EGFR Inhibitor, Andrographolide and Pifithrin-α hydrobromide were bound to the active pocket surface of EGFR, IL-6 or TP53 proteins.

**Table 3:**
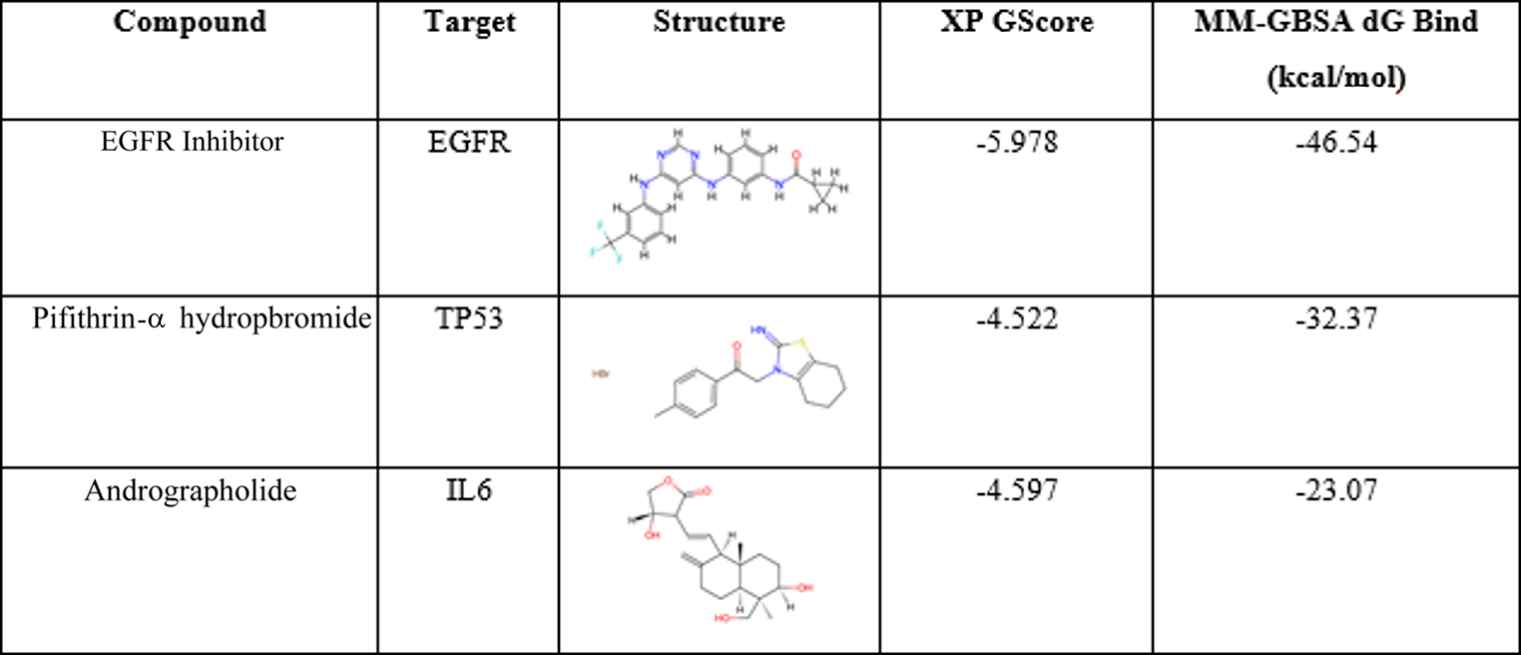
XP and MM-GBSA results for positive control drugs.

## MD analysis of the best docked ligand with target proteins

**Fig. 10:**
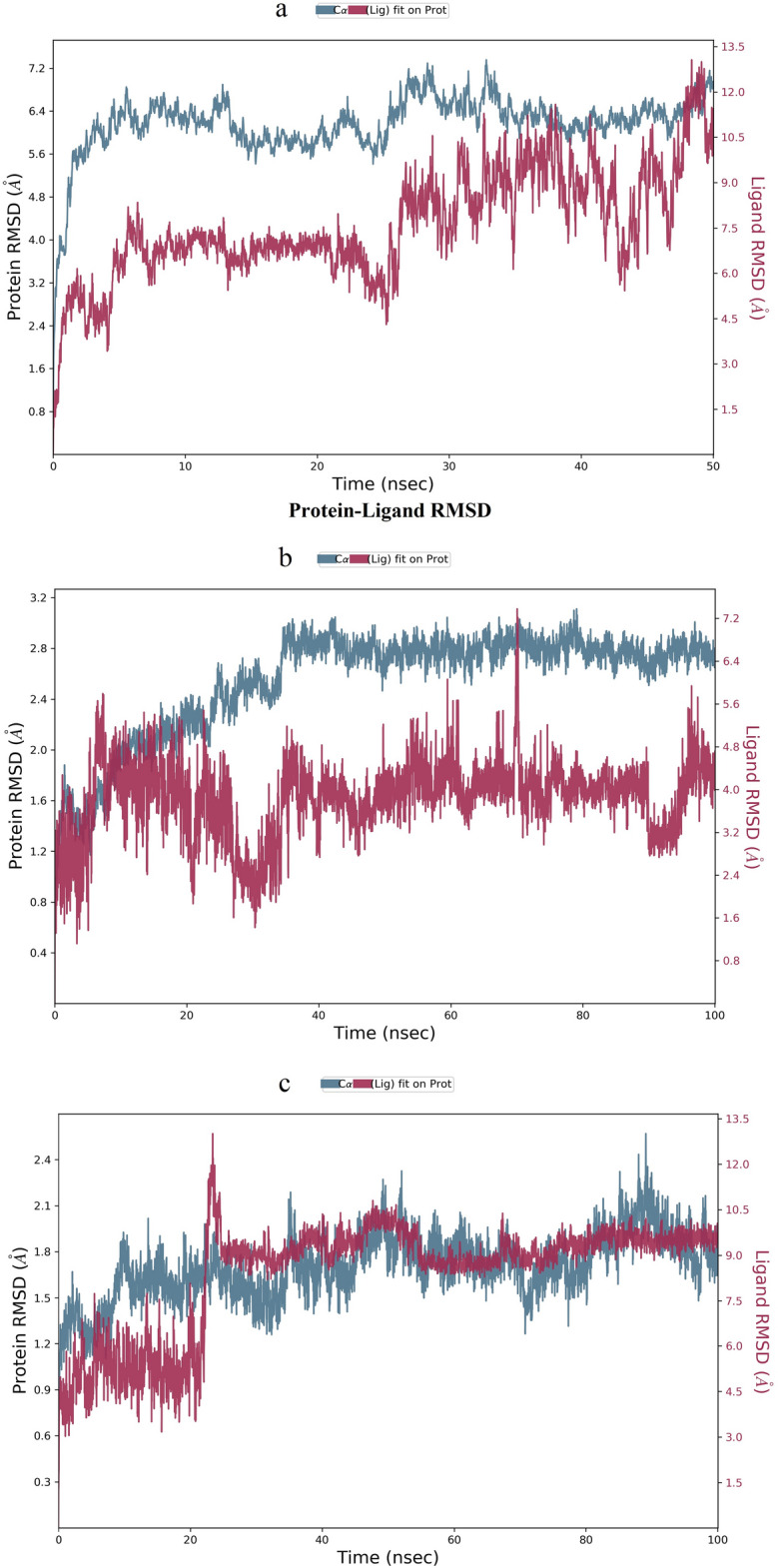
Root mean square deviation (RMSD) plot for molecular dynamics simulation: (**a**) RMSD of EGFR-QJ17; (**b**) RMSD of TP53-QJ17; (**c**) RMSD of IL6-QJ25.

To delve deeper into the protein–ligand complexes, we selected the most strongly bound active molecules for each protein and subjected them to MD simulation. Using Desmond v12, we assessed the stability of IL-6, p53, and EGFR proteins with QJ17 and QJ25, building upon the docking results and hydrogen bond interaction analysis. The root mean square deviation (RMSD) was calculated with respect to the initial structures during the 50 ns MD simulation of QJ17 and EGFR proteins to determine the stability of the complexes. Subsequently, we analyzed the MD trajectories. Figure 10 illustrates the conformational stability of RMSD against simulated time, where minor fluctuations indicate the attainment of stable conformations by all complexes. Our findings suggest that the QJ17 and EGFR proteins achieved relative stability after 30 ns, marking the system’s equilibrium. We then extended the simulation to 100 ns for QJ17 and p53 protein, observing their stability after 40 ns, upon reaching equilibrium. Subsequently, a 100 ns MD simulation of QJ25 and IL-6 was conducted, revealing their stability after 25 ns, indicating system equilibrium. In summary, the stability of these complexes confirms the validity of the docking results. Furthermore, fluctuations in temperature and pressure did not significantly influence the structural conformation.

**Fig. 11:**
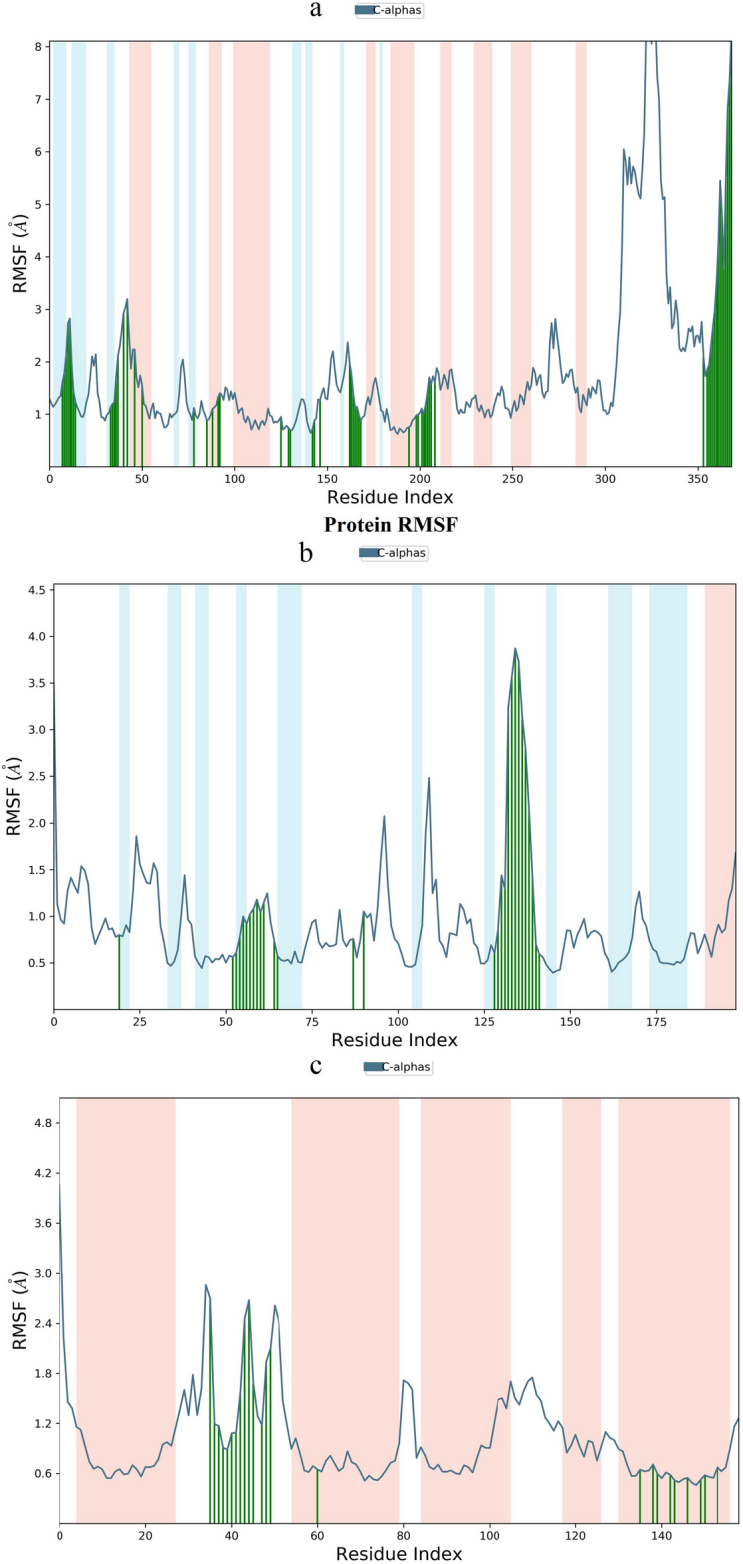
Root mean square fluctuation (RMSF) plot during molecular dynamics simulation: (**a**) RMSF of EGFR-QJ17; (**b**) RMSF of TP53-QJ17; (**c**) RMSF of IL6-QJ25.

The root mean square fluctuation (RMSF) serves as a valuable metric for characterizing local changes within the protein chain, with peaks indicating regions of pronounced fluctuation during the simulation. Figure 11 illustrates these results, highlighting the impact of QJ17 and QJ25 binding on protein structural flexibility. Upon binding of QJ17 to EGFR, the protein exhibited heightened structural flexibility in the residue region spanning 300–340 AA. Similarly, QJ17-p53 binding led to increased structural flexibility in residue regions 90–100 AA, 105–115 AA, and 125–140 AA (Fig. 11). Furthermore, following the binding of QJ25 to IL-6, the protein displayed elevated structural flexibility in residue regions 30–35 AA and 40–50 AA. These observations shed light on the localized changes induced by ligand binding, offering insights into the dynamic behavior of the protein–ligand complexes.

**Fig. 12:**
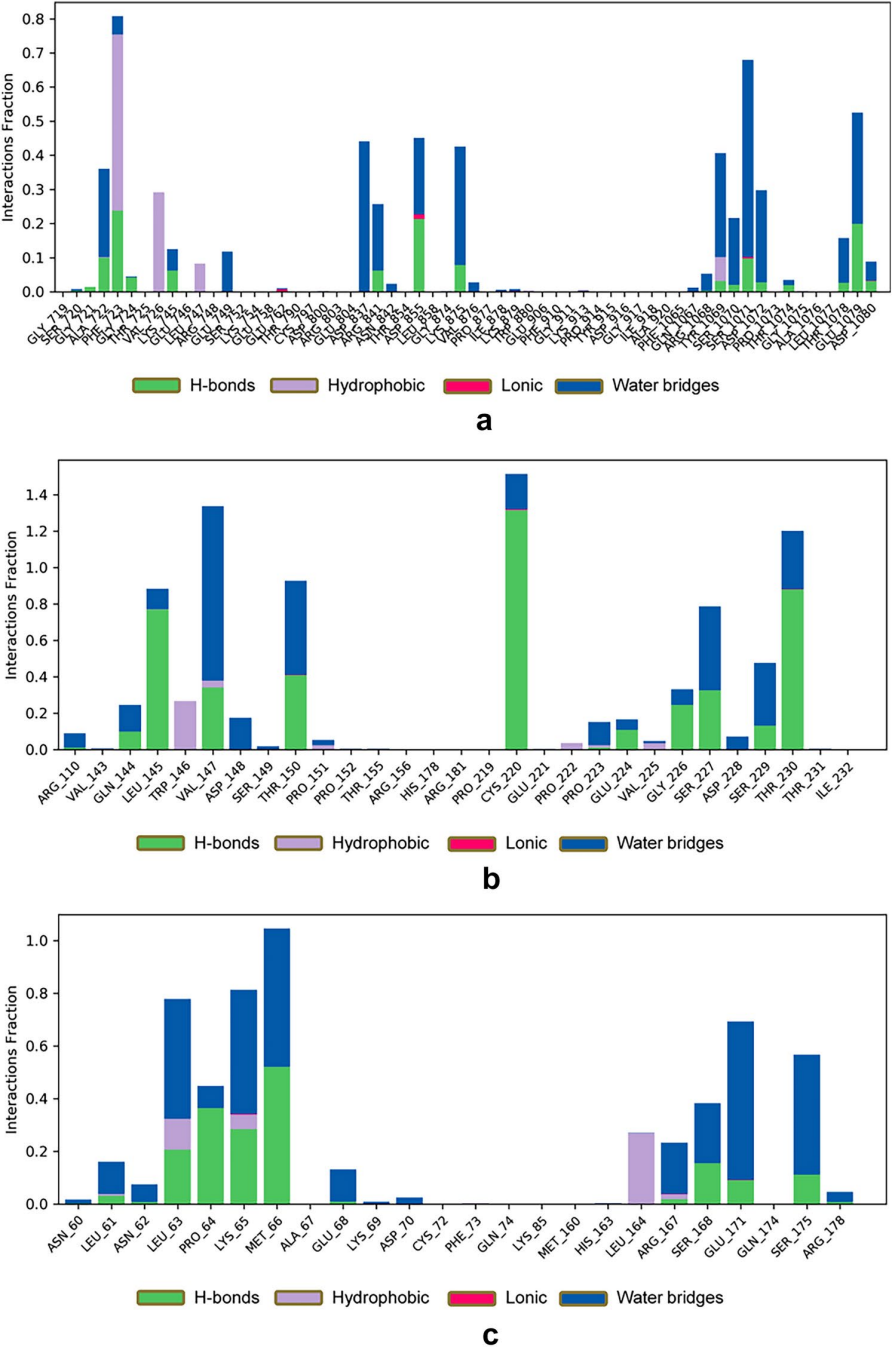
Hydrogen bonding interactions during 100 ns MD simulation of: (**a**) QJ17-EGFR residue; (**b**) QJ17-TP53 residue; (**c**) QJ25-IL6 residue.

Protein–ligand interactions, monitored throughout the simulations, can be categorized into four types: hydrogen bonding, hydrophobic interactions, ion bridges, and water bridges. As depicted in Fig. 12, key amino acids contributing to the binding of QJ17 to EGFR included ALA722, PHE723, ASP837, ASP855, LYS875, TYR1069, SER1071, and GLU1079. These interactions were predominantly facilitated by water bridges and hydrogen bonding. Additionally, Fig. 12 highlights the involvement of amino acids LEU145, VAL147, THR150, CYS200, and THR230 in the binding of QJ17 to p53, primarily through hydrogen bonding and water bridge interactions. Concerning the binding of QJ25 to IL-6, amino acids LEU63, LYS65, MET66, GLU171, and SER175 played pivotal roles, with interactions predominantly mediated by water bridges and hydrogen bonding.

**Fig. 13:**
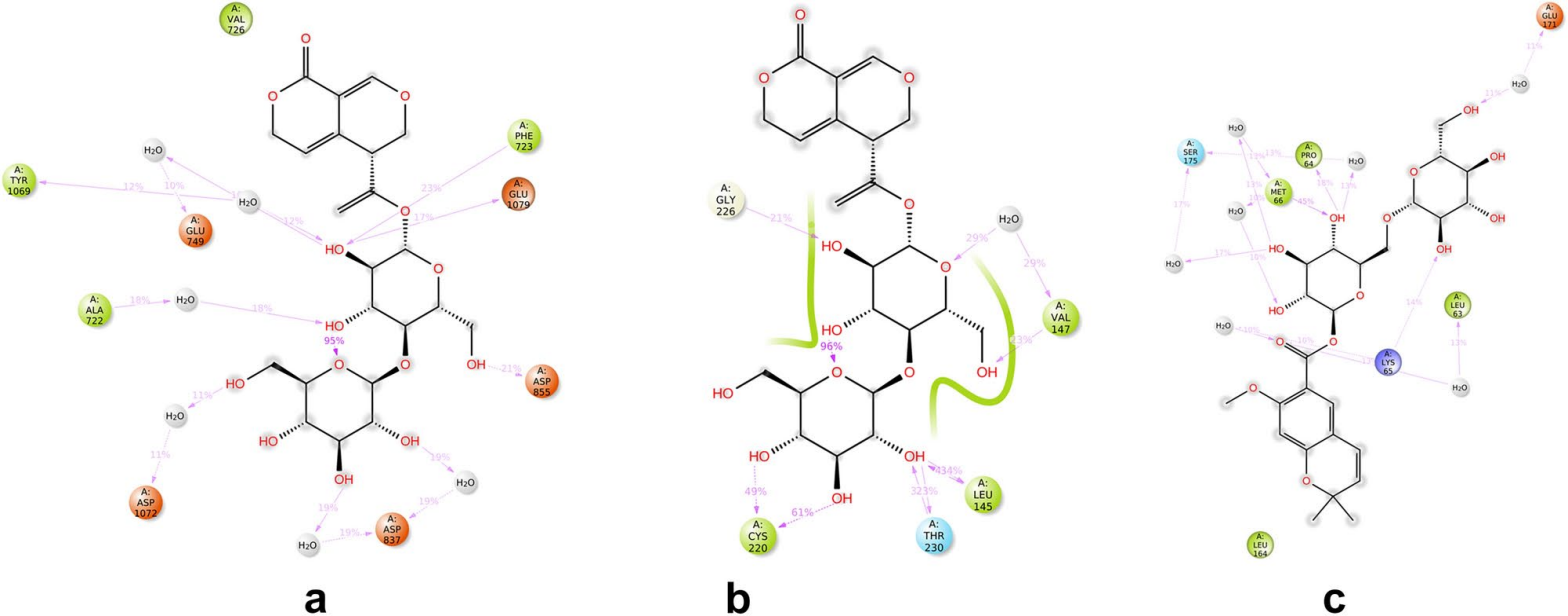
Two-dimensional diagram for the 100 ns MD simulation of: (**a**) QJ17-EGFR interaction; (**b**) QJ17-TP53 interaction; (**c**) QJ25-IL6 interaction.

Figure 13 provides detailed schematic diagrams illustrating the interactions between the compounds and protein residues, with a focus on interactions persisting for more than 10% of the simulation time (> 10 ns) in a selected locus. For QJ17 interacting with EGFR, direct hydrogen bonds were observed with amino acid residues PHE723 (23%), ASP855 (21%), and GLU1079 (17%). Indirect hydrogen bonds were formed with residues ASP837 (19%), ALA722 (18%), TYR1069 (12%), ASP1072 (11%), and GLU749 (10%) via water bridge interactions.

In the case of QJ17 binding to p53, direct hydrogen bonds were formed with amino acid residues CYS220 (61%), LEU145 (43%), THR230 (32%), and GLY226 (21%). Additionally, direct hydrogen bonds were observed with VAL147 (23%), with indirect bonds formed through water bridge interactions (29%). Furthermore, for QJ25 interacting with IL-6, direct hydrogen bonds were established with amino acid residues MET66 (45%), PRO64 (18%), and LYS65 (14%). Indirect bonds were formed with SER175 (17%), LEU63 (13%), and GLU171 (11%).

These findings elucidate the specific interactions between the compounds and protein residues, shedding light on the molecular mechanisms underlying their binding dynamics.

The binding affinities of the QJ17-EGFR, QJ17-p53, and QJ25-IL-6 complexes were analyzed using MD simulation. Hydrogen bonding, RMSD, and RMSF analyses of these docking complexes consistently demonstrated their stability throughout the simulations, suggesting their potential as inhibitors of breast cancer. In the MD simulation of the QJ17-EGFR complex, stable hydrogen bond interactions were observed with amino acid residues PHE:723, GLU:1079, ASP:855, ASP:837, ASP:1072, ALA:722, and TYR:1069. Similarly, in the QJ17-p53 MD simulation, stable hydrogen bond interactions were identified with residues GLY:226, CYS:220, THR:230, LEU:145, and VAL:147. The QJ25-IL-6 MD simulation revealed stable hydrogen bond interactions with residues GLU:171, SER:175, PRO:64, MET:66, LEU:63, and LYS:65. During the simulations, water bridges formed between specific amino acid residues and water molecules in the EGFR, p53, and IL-6 complexes. In the EGFR complex, water bridges were observed with TYR:1069, GLU:749, ALA:722, ASP:1072, and ASP:837. In the p53 complex, water bridges formed with VAL:147 and water molecules, as well as with the hydroxyl group (OH) of QJ17. Similarly, in the IL-6 complex, water bridges were established with GLU:171, SER:175, MET:66, LEU:63, and LYS:65, while MET:66 and LYS:65 additionally formed water bridges with water molecules and the hydroxyl group of QJ25. These findings underscore the stability and intricate molecular interactions within the protein–ligand complexes, further supporting their potential as therapeutic agents against breast cancer.

## Discussion

This study employs the UHPLC-ESI-Q-TOF–MS/MS technique to identify and analyze the chemical constituents of QJ. Integrating insights from network pharmacology, molecular docking, and MD simulation, we construct a comprehensive composition-target-disease network. Molecular docking and kinetic simulations of 2’-O-β-D-glucose-gentiopicrin and macrophylloside D further enhance our understanding. This multi-faceted approach sheds light on the intricate mechanisms of QJ in breast cancer treatment, emphasizing its multi-component and multi-target nature. The resulting insights provide a valuable reference for expanding the application scope of QJ and understanding its potential in the context of breast cancer therapeutics.

Recent studies have predominantly explored the antitumor properties of gentiopicroside and loganic acid, focusing primarily on their effects as iridoid terpenoids. Notably, both compounds exhibit significant anti-inflammatory effects^29,30^. In the context of cancer, emerging research highlights the inhibitory impact of gentiopicroside and loganic acid derived from QJ on various cancers, including pancreatic^31^, liver^32,33^, breast^34^, cervical^35^, ovarian^36^, and gastric cancer^37^. These findings underscore their promising potential for the development of effective antitumor drugs. This study specifically investigates the potential of 2’-O-β-D-glucose-gentiopicrin and macrophylloside D, both present in QJ, in inhibiting breast cancer. Considering the limited existing research on these components, there is a clear opportunity for novel studies in the realm of cancer treatment. Our research group aims to conduct experiments on these two constituents to elucidate their mechanisms of action in future studies.

The results of a PPI analysis indicate that QJ exhibits synergistic effects with key targets such as TP53, EGFR, and IL-6. These targets play pivotal roles in breast cancer treatment by influencing inflammatory responses, cancer proliferation, apoptosis, metastasis, and drug resistance. EGFR, a key player in cancer progression, is highly expressed in various cancers. Its involvement in regulating crucial biological characteristics such as cancer cell proliferation, metastasis, and drug resistance has been well-documented^38,39^. EGFR overexpression is particularly prevalent in approximately half of triple-negative breast cancer (TNBC) and inflammatory breast cancer (IBC) cases^40^. Studies also reveal that EGFR and its downstream pathways play a crucial role in regulating processes like epithelial-mesenchymal transformation, migration, and tumor invasion. High EGFR expression independently predicts poor prognosis in IBC. Targeting EGFR has proven effective in enhancing the chemical sensitivity of TNBC cells by reshaping the apoptotic signaling network in TNBC^41^. Currently, four generations of EGFR-targeting drugs, including Iressa, afatinib, and ocitinib, have been developed, with the first three widely used in clinical practice. However, the fourth generation is still in development, primarily suitable for advanced or metastatic non-small cell lung cancer. Stratified studies on EGFR activation in TNBC are crucial, as is the identification of biomarkers in patients responding to EGFR tyrosine kinase inhibitors (TKIs) and monoclonal antibodies (mAbs) targeting outer domains^42^.

In 1986, IL-6 was initially recognized as a B cell stimulator, promoting the differentiation of effector B cells into antibody-producing cells^43,44^. The significance of IL-6 extends beyond immune function, as it is implicated in tumor progression through inflammation, driven by the overproduction of pro-inflammatory cytokines, including IL-6 itself^45^. Chronic inflammation within the tumor microenvironment fosters tumor growth, leading to resistance against chemotherapy and radiotherapy. IL-6 cytokines are frequently overexpressed in the tumor microenvironment across various cancers, including breast cancer. Primary sources of IL-6 secretion within the tumor microenvironment include tumor cells and tumor-associated fibroblasts. Extensive research has substantiated the immunopathogenic role of IL-6 and its signaling pathway in breast cancer, influencing growth, metastasis, and drug resistance^46^. In the tumor microenvironment, inflammatory molecules are primarily released by tumor cells and stromal cells. IL-6, as a pro-inflammatory cytokine, is released by various cells, including cancer cells, playing a pivotal role in tumor cell expansion and differentiation^47–49^. Previous studies have underscored the impact of the inflammatory microenvironment on tumor growth, demonstrating that anti-inflammatory drugs can impede tumor growth in breast cancer mice models^50^. Malignant cells exhibit heightened proliferation, a process further fueled by inflammation. IL-6 influences multiple facets of tumorigenesis, regulating proliferation, apoptosis, metabolism, survival, angiogenesis, and metastasis^51^. Moreover, IL-6 is implicated in modulating therapeutic resistance, including multidrug resistance (MDR)^52^. Given its central role, targeting or blocking IL-6 and its receptors, especially in combination with other effective anticancer therapies, emerges as a promising strategy for treating breast cancer^53^.

The TP53 gene, encoding the p53 protein, stands out as the most frequently mutated gene in human cancer, with mutations observed in approximately 50% of cases. This gene plays a pivotal role in tumorigenesis, with somatic mutations occurring at a frequency of 36.1% across 20 types of cancerous tissues, surpassing all known genes^54^. The majority of TP53 mutations are missense mutations, indicating potential alterations in the structure of the p53 protein that contribute to tumorigenesis^55^. Mutant TP53 has been associated with increased transcriptional deactivation activity of NF-kB, E2F1, ETS1/ETS2, and YAP1, all known to stimulate tumor growth^56,57^. Beyond its role in transcriptional regulation, growing evidence suggests p53’s involvement in mitochondrial DNA (mtDNA) homeostasis. The mitochondrial genome (mtDNA), responsible for encoding 13 essential proteins crucial for respiration and oxidative phosphorylation, underscores the multifaceted functions of the TP53 gene^58^.

Activation of the p53 protein occurs in response to various stress signals, including DNA damage, hyperproliferative signaling, hypoxia, oxidative stress, and ribonucleotide depletion. In response, p53 inhibits cell transformation and proliferation by inducing cell cycle arrest, DNA repair, and apoptosis^59^. TP53 mutations may lead to a loss-of-function (LOF) effect, compromising cells’ anticancer protection^60^. Conversely, the gain-of-function (GOF) effect of mutant TP53 becomes crucial for sustained proliferation and survival of malignant cells.

Researchers propose that drugs capable of eliminating mutant p53 proteins could have significant therapeutic effects^61,62^. Studies have also revealed the potential for cancer treatment by targeting mutant p53 proteins, either by reducing their levels or restoring wild-type (WT) p53 function^63^. Understanding the functional abnormalities of the p53 protein and the TP53 gene is pivotal in cancer research, as they play an essential inhibitory role in cancer occurrence and development. Ongoing studies in this area hold promise for the development of new anticancer therapies, advancements in tumor diagnosis, and improved prognosis assessments.

In order to further analysis the related effects and pathways of QJ in the treatment of breast cancer, GO and KEGG pathways were enriched for potential targets.The results of the GO functional enrichment analysis showed that the core targets of QJ on breast cancer affected biological processes, including response to external stimuli, protein phosphorylation, and positive regulation of cell proliferation. This suggests that these biological processes play a crucial role in the occurrence and development of breast cancer. The KEGG pathway analysis revealed that QJ treated breast cancer by predominantly regulating the cancer pathway and HIF-1 signaling pathway.

HIF-1 stands out as an oxygen-regulated transcriptional activator, serving as a pivotal regulator of hypoxia in mammals. In the context of cancer, hypoxia, a condition induced by tumors, mirrors the physical microenvironment of most solid tumors. This hypoxic state is intricately linked to tumor occurrence and development, correlating with an increased potential for metastasis^64,65^.

Mitochondria, being major consumers of oxygen, bear the brunt of reduced oxygen availability, leading to severe damage. As the primary organelles affected by changes in oxygen levels in the microenvironment, mitochondria play a crucial role in tumor development. Consequently, genes associated with mitochondria may contain potential mediators of hypoxia-induced breast cancer progression and metastasis^66,67^.

The active and regulatory subunit of HIF-1, HIF-1α, has close ties with genes linked to estrogen production, angiogenesis, cell proliferation, and inflammation. The HIF-1 system plays a vital role in cancer cell metabolic adaptation to oxygen, promoting cancer cell proliferation, survival, and metastasis. Hypoxia induces the interaction between HIF-1α transactivation domains and coactivators. Interestingly, under non-hypoxic conditions, nitric oxide induces HIF-1α expression but inhibits its hypoxia-induced expression^68^.

Moderate increases in reactive oxygen species (ROS) levels in hypoxic cancer cells stabilize HIF-1α and mediate various tumorigenic-related signaling pathways, including AKT, NF-κB, AMPK, and Notch^69^. Consequently, HIF-1 emerges as a central regulator of cancer progression and a potential target for cancer therapy^70^.

The “cancer pathway” encompasses an array of signal transduction pathways and molecular regulatory networks crucially involved in the initiation, progression, and metastasis of cancer. Within the hsa05200 cancer pathway, essential cell signaling pathways include the regulation of the cell cycle, apoptosis, DNA damage repair, and cell migration and invasion. Maintaining the normal function of cells and preventing cancer relies heavily on the proper regulation of these signaling pathways^71,72^. However, when gene mutations or abnormalities occur within these pathways, the regulation of cell growth, proliferation, and survival may be disrupted, contributing to the development of cancer^73^. A comprehensive understanding of cancer pathways is crucial for unraveling how cancer cells evade normal regulatory mechanisms, providing valuable insights for the development of targeted therapeutic strategies. In tandem with this understanding, molecular docking and dynamics simulation studies have shed light on the stability and binding affinities of QJ17-EGFR, QJ17-TP53, and QJ25-IL-6 complexes throughout the operational process. These complexes exhibited not only stability but also optimal binding site structural conformation, suggesting their potential as novel and effective anti-breast cancer compounds derived from QJ. These findings underscore the importance of exploring natural compounds, such as those from QJ, and their interactions with key molecular targets in the cancer pathway. Such investigations not only contribute to our understanding of cancer biology but also pave the way for the development of promising anti-cancer agents.

Toxicity evaluation is one of the main steps in drug research. Toxicological tools as an effective practice in green toxicology were providing faster toxicity prediction with great prediction accuracy. Using the ProTox 3.0 prediction pipeline^74^, QJ17 has been predicted with toxicity class4 for acute oral toxicity with LD50 value of 2000 mg/kg, with a prediction accuracy of 68.07%, and QJ25 has been predicted with toxicity class5 for acute oral toxicity with LD50 value of 2260 mg/kg, with a prediction accuracy of 67.38%. These two compounds were predicted to be active for neurotoxicity and cardiotoxicity and predicted to be active for the immunotoxic, clinically toxic and nutritional toxic under the toxicological endpoints class. And QJ17 was also predicted to be active for the Blood–Brain Barrier (BBB) permeability under the toxicological endpoints class. Two MIEs endpoint-Transtyretrin (TTR) and Pregnane X receptor (PXR) were predicted as active for QJ17 and QJ25 (as reported in Figures uploaded in Supplementary Figures).

This study does have some limitations. In the present study, a comprehensive approach involving network pharmacology, molecular docking, and molecular dynamics simulations were employed to investigate the mechanism of action of 2’-O-β-D-glucose-gentiopicrin and macrophylloside D in the treatment of breast cancer. These insights provide a theoretical foundation for further isolating and studying active ingredients within QJ for breast cancer treatment, however, the depth experimental validation study have not been studied by us. Nevertheless, this does not hamper the value of our research. It provides a new thought for the study of breast cancer. Future research from our group will involve conducting experiments to validate and elucidate the anti-breast cancer mechanisms associated with QJ.

## Supplementary Information

Supplementary Information 1.

Supplementary Information 2.

Supplementary Information 3.

Supplementary Information 4.

Supplementary Information 5.

Supplementary Information 6.

Supplementary Information 7.

Supplementary Information 8.

Supplementary Information 9.

Supplementary Information 10.

Supplementary Information 11.

Supplementary Information 12.

Supplementary Information 13.

## Declarations

### Data availability

All data generated or analysed during this study are included in this published article [and its supplementary information files].
