# Supplementary material for: Mechanisms of breast cancer treatment using Gentiana robusta: evidence from comprehensive bioinformatics investigation
Source: Sci Rep. 2024 Dec 30;14:31567. doi: 10.1038/s41598-024-76063-z (PMC11686125; doi:10.1038/s41598-024-76063-z)
Supplement: Supplementary file 1 — Supplementary Information 1. [file 41598_2024_76063_MOESM1_ESM.pdf]

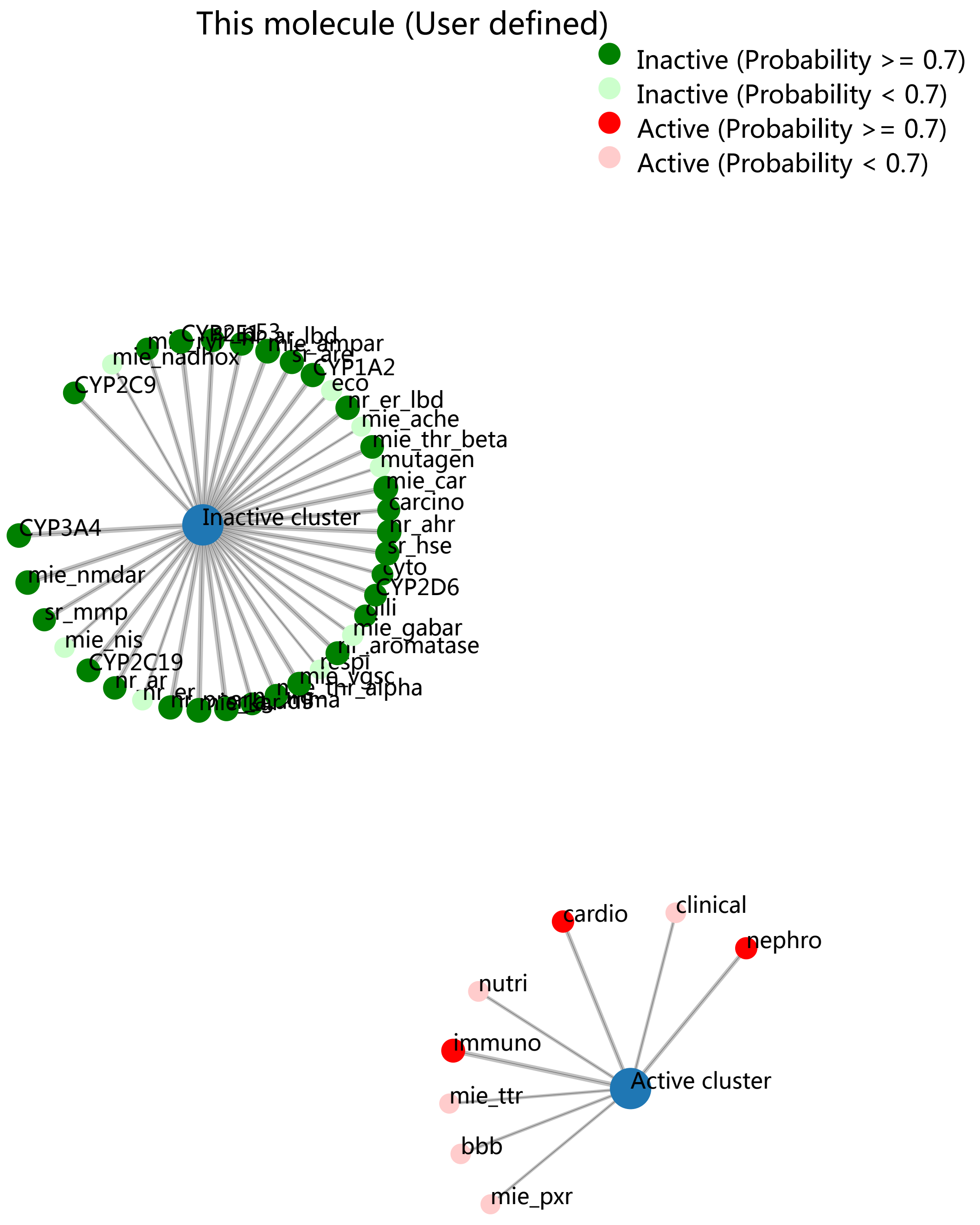
This molecule (User defined)
Inactive (Probability >= 0.7)
Inactive (Probability < 0.7)
Active (Probability >= 0.7)
Active (Probability < 0.7)
Inactive cluster
mie_nadhox
CYP2C9
mie_ampar
CYP1A2
eco
nr_er_lbd
mie_ache
mie_thr_beta
mutagen
mie_car
carcino
nr_ahr
sr_hse
cyto
CYP2D6
mie_gabar
respi
mie_vgsc
CYP3A4
mie_nmdar
sr_mmp
mie_nis
CYP2C19
nr_ar
Active cluster
cardio
clinical
nephro
nutri
immuno
mie_ttr
bbb
mie_pxr
