# Supplementary figures and images for: Mechanisms of breast cancer treatment using Gentiana robusta: evidence from comprehensive bioinformatics investigation
Source: Sci Rep. 2024 Dec 30;14:31567. doi: 10.1038/s41598-024-76063-z (PMC11686125; doi:10.1038/s41598-024-76063-z)

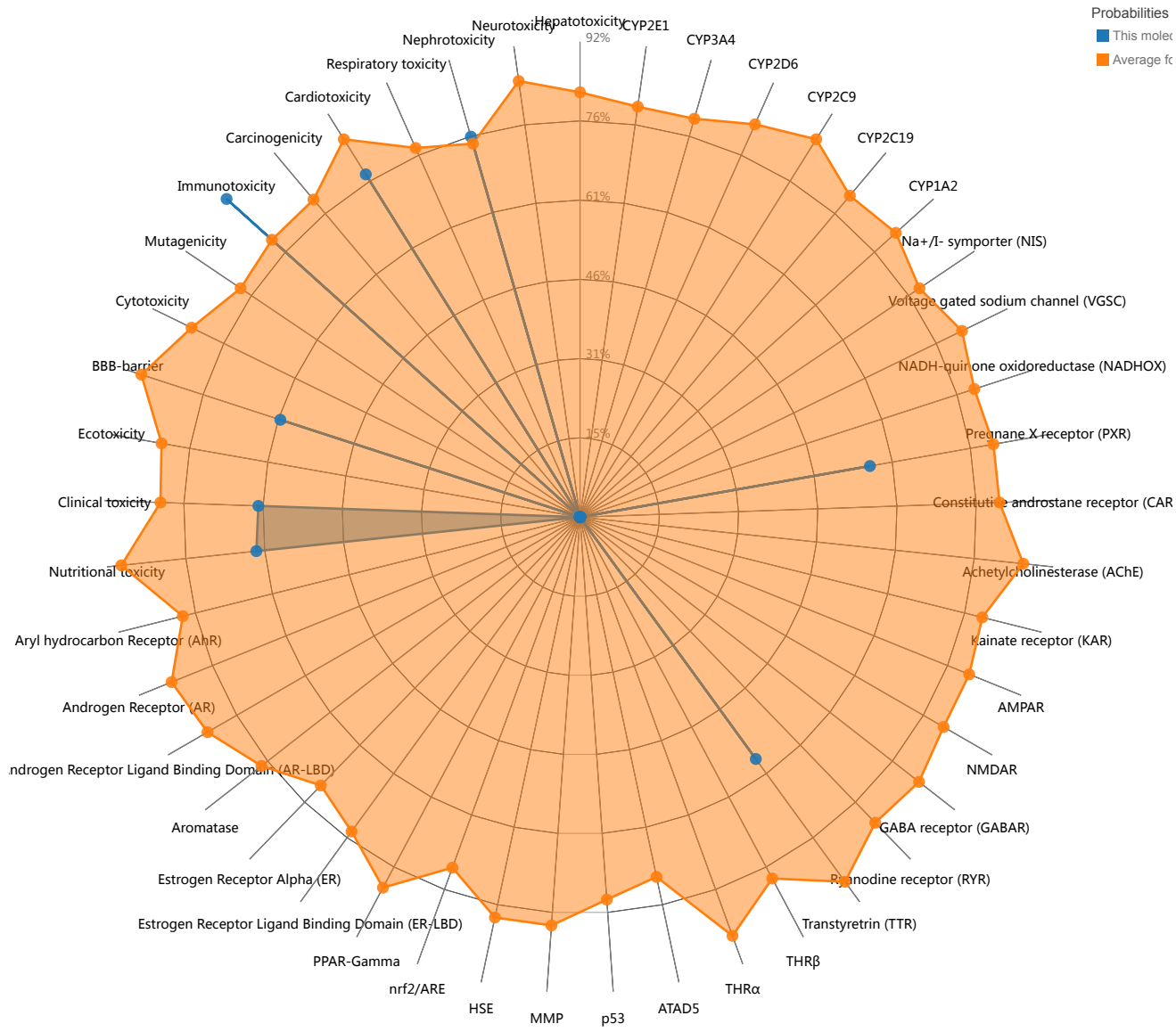

Supplement: Supplementary file 6 — Supplementary Information 6. [file 41598_2024_76063_MOESM6_ESM.pdf]

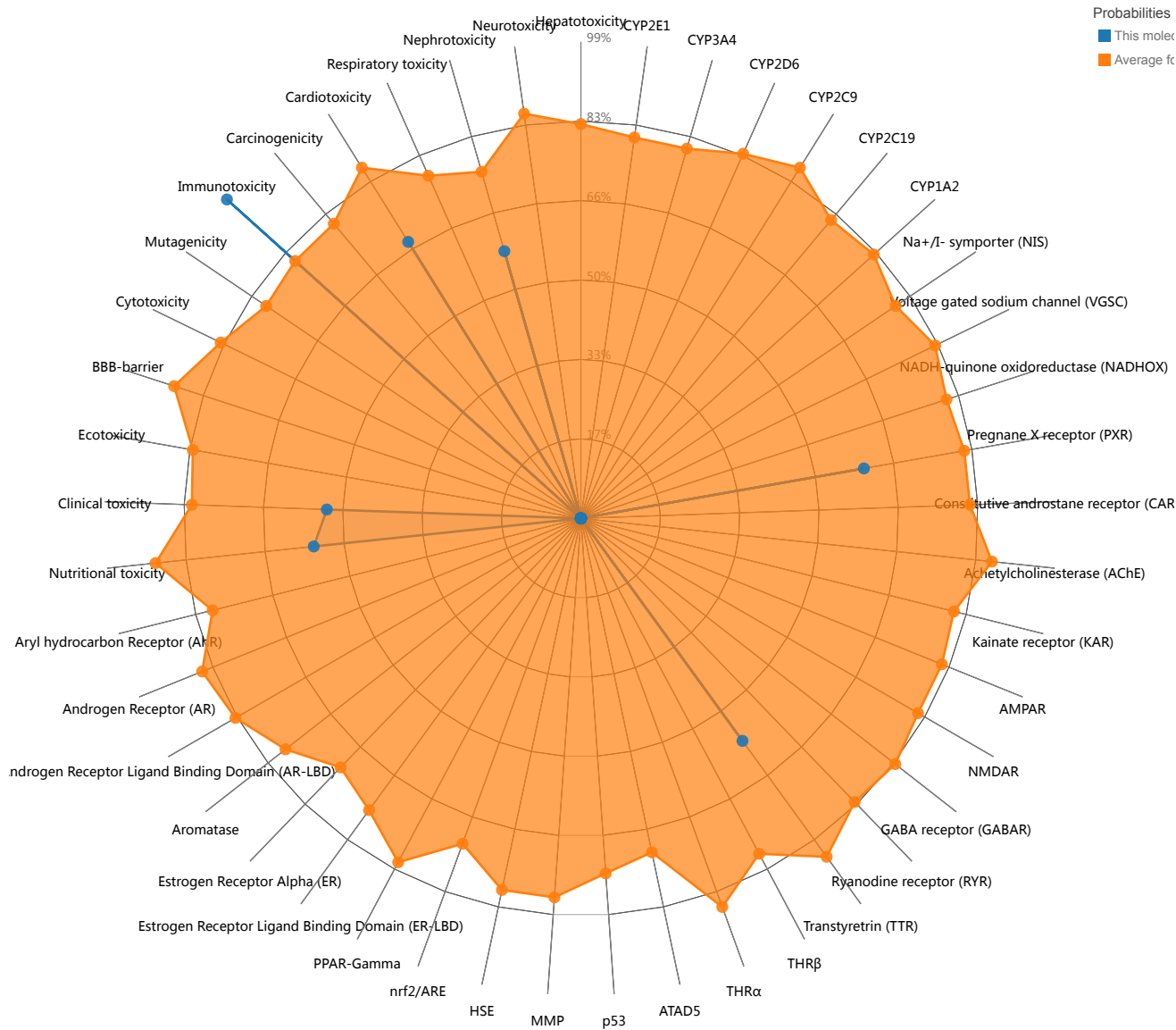

Supplement: Supplementary file 7 — Supplementary Information 7. [file 41598_2024_76063_MOESM7_ESM.pdf]
