# Supplementary material for: Mechanisms of breast cancer treatment using Gentiana robusta: evidence from comprehensive bioinformatics investigation
Source: Sci Rep. 2024 Dec 30;14:31567. doi: 10.1038/s41598-024-76063-z (PMC11686125; doi:10.1038/s41598-024-76063-z)
Supplement: Supplementary file 9 — Supplementary Information 9. [file 41598_2024_76063_MOESM9_ESM.docx]

**Table S1** Identification of chemical constituents from *Gentiana robusta* Radix by UPLC-ESI-Q-TOF-MS/MS

| No. | Precursor ion | t_R_(min) | Observed(m/z) (error, ppm) | Experimental (m/z) MS^2^  fragment ions (m/z) | Formula | Molecular Name |
| --- | --- | --- | --- | --- | --- | --- |
| QJ1 | [M−H]^-^ | 0.59 | 341.107 91 ( -1.03 ) | 323.096 54，311.096 08，281.086 19，  267.070 32，251.074 85，237.059 30，  221.064 36，179.054 22，161.043 42，  149.043 17，143.032 75，129.017 07，  119.032 51，113.022 36，101.022 24 | C_12_H_22_O_11_ | Sucrose |
| QJ2 | [M−H]^-^ | 1.41 | 375.128 62 ( -1.04 ) | 231.084 25，213.074 37，179.053 73，  169.084 59，151.073 93，125.058 11，  113.022 00，107.047 77 | C_16_H_24_O_10_ | 8-Hydroxy-10-hydroxy-swertiamarin |
| QJ3 | [M−H]^-^ | 2.10 | 375.128 35 ( -1.31 ) | 213.074 35，151.073 71，125.058 23 | C_16_H_24_O_10_ | 8-Epiloganic acid |
| QJ4 | [M−H]^-^ | 2.94 | 373.113 12 ( -0.91 ) | 211.059 52，167.069 04 | C_16_H_22_O_10_ | Secologanic acid |
| QJ5 | [M−H]^-^ | 3.26 | 375.129 00 ( -0.66 ) | 349.147 88，213.074 89，195.063 72，  169.084 99，151.073 91，133.063 11，  125.058 67，113.022 39，107.047 87 | C_16_H_24_O_10_ | Loganic acid |
| QJ6 | [M+HCOO]^-^ | 3.75 | 451.145 42 ( -0.30 ) | 243.085 32，155.032 37，141.053 34，  124.014 36，121.027 29 | C_17_H_26_O_11_ | Shanzhiside methylester |
| QJ7 | [M+HCOO]^-^ | 3.84 | 451.145 45 ( -0.27 ) | 243.085 16，155.032 44 | C_17_H_26_O_11_ | Morroniside |
| QJ8 | [M−H]^-^ | 4.35 | 389.108 36 ( -0.58 ) | 345.117 90,209.043 01,183.063 82,  165.003 19,139.001 19,121.063 53,  113.022 01,101.022 19 | C_16_H_22_O_11_ | Secologanoside |
| QJ9 | [M+HCOO]^-^ | 4.63 | 449.129 72 ( -0.35 ) | 241.069 50，127.038 12 | C_17_H_24_O_11_ | Qinjiaoside A |
| QJ10 | [M+HCOO]^-^ | 4.72 | 563.162 00 ( 0.22 ) | 265.069 41，235.058 69，193.048 31，  165.053 32，161.043 56，149.057 78，  143.032 55，131.032 92，119.032 66，  113.022 09，101.022 33， | C_22_H_30_O_14_ | 3′-O-β-D-glucopyranosyl-gentiopicroside |
| QJ11 | [M+HCOO]^-^ | 4.76 | 419.118 92 ( -0.53 ) | 419.118 39，373.112 28，355.951 29，  141.017 12 | C_16_H_22_O_10_ | Swertiamarin |
| QJ12 | [M+HCOO]^-^ | 5.06 | 565.177 54 ( 0.14 ) | 341.106 46，323.095 80，221.064 43，  195.064 12，179.053 96，151.073 97，  125.022 41 | C_22_H_32_O_14_ | Swertiapunimarin |
| QJ13 | [M+HCOO]^-^ | 5.72 | 401.108 33 ( -0.59 ) | 355.101 92，269.100 61，193.048 22，  179.053 71，149.058 28，147.042 43，  113.022 16，101.022 29 | C_16_H_20_O_9_ | Gentiopicroside |
| QJ14 | [M+H]^+^ | 5.72 | 176.053 8 ( -0.9 ) | 159.042 78，149.058 84，147.042 83，  131.048 06，121.063 78，103.053 14 | C_10_H_8_O_3_ | Erythrocentaurin |
| QJ15 | [M+H]^+^ | 5.72 | 194.064 3 ( -0.9 ) | 177.053 72，162.029 81，159.042 78，  149.058 84，147.042 83 | C_10_H_10_O_4_ | Ferulic acid |
| QJ16 | [M−H]^-^ | 5.77 | 447.149 07 ( -1.73 ) | 269.100 61 | C_19_H_28_O_12_ | 8-O-Acetylshanzhiside methyl ester |
| QJ17 | [M+HCOO]^-^ | 5.84 | 563.161 48 ( -0.30 ) | 341.107 30，315.070 13，263.075 38，  221.063 88，179.053 72，161.043 20，  143.032 27，101.022 03, | C_22_H_30_O_14_ | 2′-O-β-D-Glucopyranosyl-gentiopicroside |
| QJ18 | [M+HCOO]^-^ | 6.23 | 403.124 19 ( -0.39 ) | 195.064 09,125.022 39 | C_16_H_22_O_9_ | Sweroside |
| QJ19 | [M+HCOO]^-^ | 6.34 | 563.162 16 ( 0.38 ) | 341.107 32，161.043 58，113.022 22 | C_22_H_30_O_14_ | 4′-O-β-D-glucopyranosyl-gentiopicroside |
| QJ20 | [M−H]^-^ | 8.25 | 447.092 29 ( -0.99 ) | 357.060 00，339.049 08，327.048 91,  313.032 80，297.038 26,285.037 71 | C_21_H_20_O_11_ | Macrophylloside C-6-C-glucoside |
| QJ21 | [M+HCOO]^-^ | 9.17 | 537.160 19 ( -1.16 ) | 323.094 06，203.032 36，193.047 88，  179.054 08，161.043 55，149.057 87，  113.021 99，101.022 03 | C_24_H_28_O_11_ | 1H-2-Benzopyran-1-one,8-(b-D-glucopyranosyloxy)-3,4-dihydro-3-(3,4,5-trimethoxyphenyl)-,(3S)- |
| QJ22 | [M+HCOO]^-^ | 9.33 | 639.156 78 ( 0.13 ) | 519.113 66，499.083 86，483.110 00，  459.092 85，433.112 61，357.081 68，  315.071 08，281.064 35，251.003 76，  221.043 84，153.017 17，109.027 35 | C_27_H_30_O_15_ | Saponarin |
| QJ23 | [M−H]^-^ | 9.63 | 431.097 59 ( -0.79 ) | 413.086 14，341.065 61，311.054 64，  283.059 04，153.016 81，109.027 12 | C_21_H_20_O_10_ | Isovitexin |
| QJ24 | [M+HCOO]^-^ | 10.86 | 441.139 11 ( -1.12 ) | 315.070 84，153.017 02，152.009 28，  109.027 18 | C_19_H_24_O_9_ | Macrophylloside C |
| QJ25 | [M+HCOO]^-^ | 10.89 | 603.193 09 ( 0.00 ) | 493.133 39，477.112 98，459.111 70，  409.112 57，371.097 67，323.096 14，  263.074 60，251.089 98，233.079 76，  221.064 92，189.089 73，125.021 86,  101.022 31 | C_25_H_34_O_14_ | Macrophylloside D |
| QJ26 | [M−H]^-^ | 12.58 | 797.213 96 ( -0.63 ) | 755.203 40，635.161 94，593.151 22，  493.135 13，451.124 10，339.070 88，  315.071 06，261.038 02，177.017 02，  153.017 31 | C_35_H_42_O_21_ | Rindoside |
| QJ27 | [M+HCOO]^-^ | 13.08 | 577.154 05 ( -2.23 ) | 493.134 49，451.123 62，339.070 53，  315.070 74，297.060 0，261.038 23，  233.043 23，153.017 22 | C_26_H_28_O_12_ | Gentimacroside |
| QJ28 | [M−H]^-^ | 13.08 | 781.218 20 ( -1.45 ) | 721.196 47，566.186 75，619.166 50，  559.144 91，493.134 49，451.123 62，  339.070 53，315.070 74，261.038 23，  177.017 04，153.017 22 | C_35_H_42_O_20_ | Trifloroside |
| QJ29 | [M+H]^+^ | 13.85 | 235.095 4 ( -1.1 ) | 219.062 68，217.084 51，205.047 81，  202.060 50，187.037 36，174.065 88，  159.042 48 | C_13_H_14_O_4_ | 2-Methoxyanofinic acid |
| QJ30 | [M+HCOO]^-^ | 15.88 | 549.342 17 ( -1.13 ) | 471.306 22,391.297 32,374.243 09 | C_30_H_48_O_6_ | 1*β*，2*α*，3*α*，24-tetrahydroxyolean-12-en-28-oic acid |
| QJ31 | [M−H]^-^ | 16.92 | 487.342 25 ( -0.66 ) | 453.299 58 | C_30_H_48_O_5_ | 2*α*，3*β*，24-tetrahydroxyurs-12-en-28-oic acid |
| QJ32 | [M−H]^-^ | 16.98 | 501.321 29 ( -0.87 ) | 343.225 88， | C_30_H_46_O_6_ | 1*α*，2*α*，3*β*，24-tetrahydroxyursa-12,20(30)-dien-28-oic acid |
| QJ33 | [M−H]^-^ | 18.00 | 487.342 66 ( -0.25 ) | 453.299 90，277.214 99 | C_30_H_48_O_5_ | 3*β*,6*α*,24-trihydroxyolean-12-en-28-oic acid |
| QJ34 | [M−H]^-^ | 21.28 | 471.347 22 ( -0.76 ) | 455.351 03 | C_30_H_48_O_4_ | 3,24-dihydroxyurs-12-en-28-oic acid |
| QJ35 | [M−H]^-^ | 22.83 | 455.352 55 ( -0.53 ) | 437.339 88 | C_30_H_48_O_3_ | Oleanic acid |
| QJ36 | [M−H]^-^ | 23.45 | 455.352 05 ( -1.03 ) | 395.329 18,369.314 44 | C_30_H_48_O_3_ | Ursolic acid |
| QJ37 | [M+H]^+^ | 24.6 | 413.375 9 ( -1.9 ) | 395.365 32，337.284 34，245.224 74，  229.193 27，217.193 56，191.177 97，  189.161 99，163.146 65，149.131 12，  121.099 86，109.099 81，107.084 20 | C_29_H_48_O | Stigmasterol |
| QJ38 | [M+H]^+^ | 25.96 | 441.373 3 ( 0.6 ) | 423.362 26，411.362 30，297.255 70，  255.209 21，229.193 70，215.177 93，  203.178 11，177.162 43，149.131 27，  121.099 99 | C_30_H_48_O2 | Roburic acid |
| QJ39 | [M−H]^-^ | 26.93 | 471.347 45 ( -0.53 ) | 427.356 63，409.346 39，393.314 59，  125.094 41 | C_30_H_48_O_4_ | Corosolic acid |
