# Supplementary material for: Mechanisms of breast cancer treatment using Gentiana robusta: evidence from comprehensive bioinformatics investigation
Source: Sci Rep. 2024 Dec 30;14:31567. doi: 10.1038/s41598-024-76063-z (PMC11686125; doi:10.1038/s41598-024-76063-z)
Supplement: Supplementary file 10 — Supplementary Information 10. [file 41598_2024_76063_MOESM10_ESM.doc]

**Table S2** Topological analysis of the PPI network based on three centrality algorithms

| Target name | Degree centrality | Target name | Betweenness centrality | Target name | Closeness centrality |
| --- | --- | --- | --- | --- | --- |
| **TP53** | **75** | **TP53** | **0.185069** | **TP53** | **0.520343** |
| **EGFR** | **56** | **IL6** | **0.088949** | **EGFR** | **0.492901** |
| **IL6** | **55** | **EGFR** | **0.0818** | **IL6** | **0.486** |
| HSP90AA1 | 50 | SRC | 0.071918 | SRC | 0.483101 |
| TNF | 49 | PTGS2 | 0.0643 | GAPDH | 0.481188 |
| STAT3 | 48 | TNF | 0.060704 | STAT3 | 0.478346 |
| SRC | 45 | HSP90AA1 | 0.056621 | HSP90AA1 | 0.472763 |
| GAPDH | 40 | ESR1 | 0.053633 | TNF | 0.47093 |
| MAPK1 | 38 | GAPDH | 0.051498 | MAPK1 | 0.461977 |
| CASP3 | 36 | STAT3 | 0.046388 | ESR1 | 0.453358 |
| ESR1 | 35 | PRKCB | 0.045701 | MAPK3 | 0.453358 |
| MAPK3 | 35 | APP | 0.045597 | CASP3 | 0.450835 |
| CCND1 | 33 | MAPK1 | 0.042283 | PTGS2 | 0.448339 |
| PTGS2 | 33 | AR | 0.041884 | MMP9 | 0.446691 |
| CXCL8 | 28 | PPARG | 0.038649 | CCND1 | 0.443431 |
| MMP9 | 27 | ITGB3 | 0.036797 | RELA | 0.43705 |
| TLR4 | 27 | MAPK3 | 0.032236 | PPARG | 0.43705 |
| ITGB1 | 27 | SLC6A3 | 0.031651 | TLR4 | 0.433155 |
| PIK3CA | 26 | ITGB1 | 0.031267 | CXCL8 | 0.429329 |
| ICAM1 | 26 | SLC6A4 | 0.028803 | AR | 0.427817 |
| PARP1 | 25 | CASP3 | 0.026004 | APP | 0.425569 |
| RELA | 25 | CXCL8 | 0.024411 | ICAM1 | 0.424825 |
| MDM2 | 24 | SLC2A1 | 0.023341 | PIK3CA | 0.421144 |
| APP | 24 | PPARA | 0.022953 | CASP8 | 0.418966 |
| CDK2 | 24 | F2 | 0.022634 | FGF2 | 0.417526 |
| CDK4 | 23 | TRPV1 | 0.02187 | ITGB1 | 0.41681 |
| CDK1 | 23 | AGTR1 | 0.019907 | HRAS | 0.415385 |
| PPARG | 23 | TYMS | 0.019501 | GSK3B | 0.414676 |
| BCL2L1 | 22 | SREBF2 | 0.017364 | MDM2 | 0.413969 |
| GSK3B | 22 | DPP4 | 0.017254 | BCL2L1 | 0.413265 |
| AR | 22 | CCND1 | 0.016844 | PARP1 | 0.411864 |
| CCNB1 | 21 | HK2 | 0.016653 | IL2 | 0.409781 |
| FGF2 | 21 | CYP1A1 | 0.016648 | PGR | 0.409091 |
| HRAS | 20 | ACE | 0.014564 | NR3C1 | 0.408403 |
| CASP8 | 20 | XDH | 0.014546 | MET | 0.408403 |
| MMP2 | 20 | IL2 | 0.012817 | MMP2 | 0.405 |
| CCNA2 | 19 | HSPA5 | 0.012609 | HSPA5 | 0.404326 |
| CYP1A1 | 18 | PIK3CA | 0.012501 | HSPA8 | 0.401653 |
| PTPN11 | 18 | HSPA8 | 0.012459 | PPARA | 0.400329 |
| IL2 | 17 | TLR4 | 0.012395 | MAP2K1 | 0.399671 |
| CHEK1 | 17 | CYP1A2 | 0.011962 | NFE2L2 | 0.399015 |
| MET | 17 | SLC5A2 | 0.011691 | PRKCD | 0.398361 |
| CYP1A2 | 16 | PTGS1 | 0.011567 | ESR2 | 0.395122 |
| PGR | 16 | TYMP | 0.011171 | CDK4 | 0.393841 |
| ITGB2 | 16 | ICAM1 | 0.010981 | CDK1 | 0.390048 |
| FYN | 15 | NR3C1 | 0.010734 | CDK2 | 0.388179 |
| ACE | 15 | CYP2A6 | 0.010622 | MCL1 | 0.386328 |
| PRKCD | 15 | LGALS3 | 0.010599 | CCNB1 | 0.386328 |
| MAP2K1 | 15 | MMP9 | 0.010469 | SLC2A1 | 0.386328 |
| CYP2C9 | 14 | ADA | 0.010401 | ABL1 | 0.385714 |
| ABL1 | 14 | AKR1C3 | 0.010362 | FYN | 0.385714 |
| MCL1 | 14 | CYP19A1 | 0.009963 | PTPN11 | 0.385103 |
| HSPA8 | 14 | GSK3B | 0.009559 | ITGB3 | 0.383281 |
| NR3C1 | 14 | HK1 | 0.00886 | CASP1 | 0.382075 |
| CCNA1 | 14 | FAAH | 0.008801 | ITGB2 | 0.382075 |
| ITGB3 | 14 | CDA | 0.008513 | ACE2 | 0.379095 |
| ACE2 | 13 | RELA | 0.008435 | IGFBP3 | 0.379095 |
| AKR1C3 | 13 | CDK4 | 0.008334 | PRKCB | 0.376161 |
| AURKA | 13 | ADRA1D | 0.00823 | ACE | 0.373846 |
| TOP2A | 13 | CA9 | 0.00823 | PTPN6 | 0.373272 |
| PTPN6 | 13 | RORC | 0.00823 | AURKA | 0.372699 |
| MMP3 | 13 | PTPN11 | 0.007975 | CDK9 | 0.372699 |
| PPARA | 13 | CDK1 | 0.007664 | MMP3 | 0.372129 |
| CYP2A6 | 12 | PNP | 0.007565 | NOS2 | 0.370992 |
| CYP19A1 | 12 | ACE2 | 0.007448 | CHEK1 | 0.370427 |
| PRKCB | 12 | HRAS | 0.007376 | CD81 | 0.370427 |
| CDC25A | 12 | PARP1 | 0.007116 | MMP1 | 0.369863 |
| MMP1 | 12 | REN | 0.007023 | AGTR1 | 0.366516 |
| CYP2C19 | 11 | NFE2L2 | 0.006959 | CCNA2 | 0.365964 |
| DPP4 | 11 | PTGES | 0.006745 | LGALS3 | 0.365964 |
| HSPA5 | 11 | NR3C2 | 0.006689 | ITGA4 | 0.364318 |
| PTGS1 | 11 | GSTP1 | 0.00652 | CSNK2A2 | 0.364318 |
| NFE2L2 | 11 | DRD1 | 0.006251 | SREBF2 | 0.364318 |
| SELP | 11 | SIGMAR1 | 0.006124 | REN | 0.363772 |
| GSTP1 | 11 | CCNB1 | 0.005931 | MGMT | 0.363772 |
| REN | 10 | NR1H4 | 0.00588 | CSNK2A1 | 0.363229 |
| SELE | 10 | FABP5 | 0.005683 | ITGAL | 0.363229 |
| NR3C2 | 10 | SRD5A2 | 0.005179 | HSPA1A | 0.362687 |
| F2 | 10 | CYP2C9 | 0.005176 | MIF | 0.362687 |
| CYP17A1 | 10 | FOLH1 | 0.005045 | THRB | 0.362687 |
| RPA1 | 10 | ALOX5 | 0.005013 | CYP1A2 | 0.362146 |
| TYMS | 10 | CDK2 | 0.00496 | PTPN1 | 0.361607 |
| CDC25B | 10 | G6PD | 0.004893 | NR3C2 | 0.36107 |
| WEE1 | 10 | NR1I2 | 0.004723 | WEE1 | 0.360534 |
| ITGA4 | 10 | FGF2 | 0.00465 | HK2 | 0.36 |
| F3 | 10 | F3 | 0.004644 | KDM6B | 0.36 |
| ITGAL | 10 | MMP2 | 0.004374 | TERT | 0.359467 |
| CYP2D6 | 9 | EPHA2 | 0.004188 | SELP | 0.359467 |
| XDH | 9 | MDM2 | 0.004148 | GSTP1 | 0.359467 |
| MME | 9 | DRD4 | 0.004087 | KDM4A | 0.358407 |
| AGTR1 | 9 | PGR | 0.003987 | G6PD | 0.357879 |
| SLC6A3 | 9 | PRKCD | 0.00385 | XDH | 0.357353 |
| CASP1 | 9 | HPRT1 | 0.003842 | SELE | 0.357353 |
| TLR9 | 9 | BCL2L1 | 0.003803 | CYP1A1 | 0.357353 |
| CDK9 | 9 | CDK9 | 0.003722 | TLR9 | 0.356828 |
| ITGAV | 9 | CNR1 | 0.003714 | TYMS | 0.356305 |
| ESR2 | 9 | CYP17A1 | 0.003689 | BTK | 0.356305 |
| ITGA2B | 9 | DRD2 | 0.003631 | F3 | 0.356305 |
| NR1I2 | 8 | CYP2D6 | 0.003615 | TOP2A | 0.355263 |
| EPHA2 | 8 | CCNA2 | 0.003432 | APEX1 | 0.354745 |
| PSEN1 | 8 | IDO1 | 0.003395 | CDC25A | 0.354745 |
| SLC6A4 | 8 | SLC6A2 | 0.003335 | IDO1 | 0.354745 |
| CYP1B1 | 8 | MME | 0.003253 | FOLH1 | 0.354227 |
| HK2 | 8 | PTGFR | 0.00305 | EPHA2 | 0.353712 |
| AKR1C1 | 8 | ITGB2 | 0.00303 | F2 | 0.353712 |
| SRD5A1 | 8 | MGAM | 0.002922 | PTPN2 | 0.353712 |
| PTGES | 8 | ESR2 | 0.002823 | ITGA2B | 0.353712 |
| ALOX15 | 8 | HSD11B1 | 0.002703 | PTGS1 | 0.353198 |
| ALOX5 | 8 | ADORA1 | 0.002638 | CCNA1 | 0.352685 |
| BTK | 8 | TK1 | 0.002608 | MMP7 | 0.352685 |
| SLC2A1 | 8 | PTPN1 | 0.002546 | PRKCE | 0.352174 |
| PRKCQ | 8 | CYP2C19 | 0.002425 | CYP19A1 | 0.351156 |
| CD81 | 8 | ITGA2B | 0.002407 | FGF1 | 0.351156 |
| HSD11B1 | 8 | HMGCR | 0.002396 | ITGAV | 0.350649 |
| GSTA1 | 8 | MAP2K1 | 0.002287 | LGALS4 | 0.350649 |
| LGALS3 | 8 | PPP1CA | 0.002272 | RPA1 | 0.34964 |
| FGFR1 | 8 | ABCB1 | 0.002156 | PRKCQ | 0.349138 |
| SREBF2 | 8 | PRKCE | 0.001987 | AHR | 0.347639 |
| SLC5A2 | 7 | FYN | 0.001908 | TRPV1 | 0.346154 |
| HK1 | 7 | MET | 0.001871 | HSF1 | 0.346154 |
| SRD5A2 | 7 | ACHE | 0.001815 | CDK8 | 0.345661 |
| TK1 | 7 | HSD11B2 | 0.001797 | TOP1 | 0.34517 |
| IGFBP3 | 7 | CHEK1 | 0.001772 | NR1I3 | 0.34517 |
| PTPN1 | 7 | CASP8 | 0.001711 | FGFR1 | 0.34517 |
| MGAM | 6 | SQLE | 0.001666 | RORC | 0.34517 |
| G6PD | 6 | TTR | 0.001664 | PTGER4 | 0.34517 |
| PNP | 6 | ALOX15 | 0.001628 | DUSP1 | 0.344681 |
| CCNC | 6 | MMP3 | 0.001574 | POLB | 0.343706 |
| TYMP | 6 | AHR | 0.001562 | SLC5A1 | 0.343706 |
| TERT | 6 | PSEN1 | 0.001531 | MME | 0.34322 |
| DRD2 | 6 | THRB | 0.001486 | OGA | 0.342736 |
| TRPV1 | 6 | AURKA | 0.001462 | RPS6KA3 | 0.341772 |
| COL18A1 | 6 | F10 | 0.001343 | CYP2D6 | 0.340336 |
| CSNK2A1 | 6 | TOP2A | 0.001284 | PSEN1 | 0.33986 |
| HSD11B2 | 6 | BTK | 0.001231 | CYP2C9 | 0.339385 |
| IDO1 | 6 | ABL1 | 0.001204 | MMP13 | 0.339385 |
| PRKCE | 6 | PTPN6 | 0.001142 | ABCB1 | 0.338912 |
| FGF1 | 6 | SELP | 0.001131 | HPRT1 | 0.338912 |
| HSF1 | 6 | AKR1C1 | 0.001126 | CXCR1 | 0.338912 |
| HSPA1A | 6 | PTGER4 | 0.001119 | ADAM17 | 0.337969 |
| NOS2 | 6 | ADRA2A | 0.001074 | ALOX15 | 0.337969 |
| MMP7 | 6 | BCHE | 0.001032 | HK1 | 0.337032 |
| ABCB1 | 5 | HTR2A | 0.001021 | PLA2G1B | 0.336565 |
| HMGCR | 5 | FABP4 | 0.000964 | MMP12 | 0.3361 |
| ACHE | 5 | CTSD | 0.000933 | MAP3K8 | 0.335635 |
| ADAM17 | 5 | SRD5A1 | 0.000898 | CYP2C19 | 0.33425 |
| PPP1CA | 5 | KDM6B | 0.000897 | DPP4 | 0.33425 |
| ADA | 5 | PLA2G1B | 0.000896 | CTSD | 0.332421 |
| DRD1 | 5 | TLR9 | 0.000879 | PTPRG | 0.330612 |
| AHR | 5 | CSNK2A1 | 0.000804 | CYP17A1 | 0.329715 |
| AKR1C2 | 5 | CCNA1 | 0.000778 | COL18A1 | 0.329715 |
| PLA2G1B | 5 | NR1I3 | 0.000768 | ACHE | 0.329268 |
| APEX1 | 5 | CD81 | 0.000767 | NCSTN | 0.329268 |
| KDM3A | 5 | KDM3A | 0.000763 | PTGFR | 0.328378 |
| CDA | 5 | AKR1B1 | 0.000762 | KDM3A | 0.327052 |
| FABP4 | 5 | AKR1B10 | 0.000762 | FABP4 | 0.326174 |
| DRD4 | 5 | PRKCQ | 0.000761 | CYP27B1 | 0.326174 |
| HTR2A | 5 | SLC5A1 | 0.000731 | PTGES | 0.325737 |
| KDM6B | 5 | GSTA1 | 0.000721 | KDM5B | 0.324433 |
| RPS6KA3 | 5 | CASP1 | 0.000665 | HSD11B1 | 0.324 |
| FABP5 | 5 | CYP1B1 | 0.000644 | PIM1 | 0.324 |
| THRB | 5 | SELE | 0.000594 | PPP1CA | 0.323569 |
| PTGER4 | 5 | ITGA4 | 0.000592 | ALOX5 | 0.323138 |
| HPRT1 | 4 | NCSTN | 0.000576 | GSTA1 | 0.321004 |
| ADRA2A | 4 | FGFR1 | 0.000575 | MKNK2 | 0.32058 |
| PTGFR | 4 | MMP1 | 0.000566 | MKNK1 | 0.32058 |
| AKR1B1 | 4 | TRPM8 | 0.000536 | DUSP16 | 0.320158 |
| AKR1B10 | 4 | MMP7 | 0.000523 | CDC25B | 0.319737 |
| CTSD | 4 | MGMT | 0.000505 | SLC5A2 | 0.319317 |
| NCSTN | 4 | ITGAV | 0.000446 | NR1H4 | 0.319317 |
| FOLH1 | 4 | ITGAL | 0.000444 | FABP5 | 0.319317 |
| KDM4A | 4 | ADRA2C | 0.000416 | SCD | 0.319317 |
| CDK8 | 4 | LGALS4 | 0.000414 | HMGCR | 0.317647 |
| TOP1 | 4 | KDM4A | 0.000396 | SHBG | 0.317647 |
| CNR1 | 4 | CDK8 | 0.000392 | SRD5A2 | 0.317232 |
| FAAH | 4 | AKR1C2 | 0.000385 | PPARD | 0.315995 |
| MMP13 | 4 | WEE1 | 0.000342 | PTGER3 | 0.315995 |
| CSNK2A2 | 4 | FGF1 | 0.000341 | CYP1B1 | 0.315584 |
| NR1I3 | 4 | IGFBP3 | 0.000315 | PTGER2 | 0.315584 |
| DRD3 | 4 | ADAM17 | 0.000276 | PNP | 0.315175 |
| PTPN2 | 4 | COL18A1 | 0.000237 | CYP2A6 | 0.314767 |
| MGMT | 4 | APEX1 | 0.000236 | HSD11B2 | 0.314767 |
| PPARD | 4 | HSPA1A | 0.000231 | BCHE | 0.311938 |
| SQLE | 4 | HSF1 | 0.000222 | TTR | 0.31114 |
| SCD | 4 | MMP13 | 0.00022 | LTB4R | 0.307595 |
| RORC | 4 | RPA1 | 0.000218 | AKR1C3 | 0.306045 |
| MMP12 | 4 | CDC25A | 0.000206 | FFAR1 | 0.304511 |
| SLC6A2 | 3 | MCL1 | 0.000205 | SORT1 | 0.30375 |
| ADRA2C | 3 | GPBAR1 | 0.000195 | SIGMAR1 | 0.303371 |
| POLB | 3 | TERT | 0.000192 | KDM4C | 0.302993 |
| TTR | 3 | SCD | 0.000146 | PSEN2 | 0.29963 |
| BCHE | 3 | RPS6KA3 | 0.000136 | MGAM | 0.298526 |
| PSEN2 | 3 | LTB4R | 0.000135 | NR1I2 | 0.29743 |
| CXCR1 | 3 | MMP12 | 0.000114 | THRA | 0.29743 |
| SHBG | 3 | CCNC | 8.15E-05 | TK1 | 0.297066 |
| SLC5A1 | 3 | ACP1 | 7.64E-05 | LGALS8 | 0.296703 |
| SIGMAR1 | 3 | CDC25B | 6.59E-05 | ADA | 0.295981 |
| DUSP1 | 3 | CSNK2A2 | 5.94E-05 | CCNC | 0.293833 |
| NR1H4 | 3 | DRD3 | 4.65E-05 | SQLE | 0.288599 |
| F10 | 3 | CHRNA4 | 4.16E-05 | SLC6A4 | 0.288256 |
| TRPM8 | 3 | ALOX5AP | 4.05E-05 | TYMP | 0.287915 |
| HTR1B | 3 | NOS2 | 3.31E-05 | TUBB3 | 0.286895 |
| MAP3K8 | 3 | CXCR1 | 1.63E-05 | SLC6A3 | 0.285882 |
| LGALS4 | 3 | PPARD | 8.50E-06 | AKR1B1 | 0.284877 |
| MKNK2 | 3 | MAP3K8 | 5.67E-06 | AKR1B10 | 0.284877 |
| MKNK1 | 3 | HTR1B | 4.86E-06 | AKR1C1 | 0.283217 |
| PTGER2 | 3 | ADRA1A | 0 | TRPM8 | 0.282887 |
| PTGER3 | 3 | POLB | 0 | F10 | 0.279954 |
| ACP1 | 2 | SORT1 | 0 | CA9 | 0.279632 |
| ADORA1 | 2 | PSEN2 | 0 | ACP1 | 0.27931 |
| ADRA1D | 2 | KDM5B | 0 | PTPRF | 0.27867 |
| LTB4R | 2 | KDM4C | 0 | SRD5A1 | 0.278032 |
| ALOX5AP | 2 | CA12 | 0 | CDA | 0.277714 |
| SORT1 | 2 | CES2 | 0 | TYR | 0.273649 |
| KDM5B | 2 | TOP1 | 0 | ADRA1D | 0.269103 |
| KDM4C | 2 | SHBG | 0 | FBP1 | 0.268508 |
| CA9 | 2 | CYP27B1 | 0 | LGALS9 | 0.268212 |
| CHRNA4 | 2 | DHODH | 0 | CNR1 | 0.267327 |
| DUSP16 | 2 | PREP | 0 | NR1H2 | 0.267327 |
| EDNRA | 2 | DUSP1 | 0 | AKR1C2 | 0.266447 |
| EDNRB | 2 | DUSP16 | 0 | FAAH | 0.265574 |
| FBP1 | 2 | EDNRA | 0 | EDNRA | 0.261853 |
| GPBAR1 | 2 | EDNRB | 0 | EDNRB | 0.261853 |
| MIF | 2 | PTPN2 | 0 | TBXAS1 | 0.26129 |
| LGALS8 | 2 | PTPRG | 0 | GPBAR1 | 0.257415 |
| THRA | 2 | FABP3 | 0 | RORA | 0.256871 |
| PTPRF | 2 | FBP1 | 0 | DRD2 | 0.254184 |
| ADRA1A | 1 | FFAR1 | 0 | ALOX5AP | 0.251293 |
| CA12 | 1 | TUBB3 | 0 | PREP | 0.250774 |
| CES2 | 1 | MIF | 0 | SLC6A2 | 0.25 |
| CYP27B1 | 1 | LGALS8 | 0 | ADRA2A | 0.238469 |
| DHODH | 1 | LGALS9 | 0 | DRD1 | 0.238002 |
| PREP | 1 | MKNK2 | 0 | HTR2A | 0.237537 |
| PTPRG | 1 | MKNK1 | 0 | ADORA1 | 0.235922 |
| FABP3 | 1 | NR1H2 | 0 | DRD4 | 0.23433 |
| FFAR1 | 1 | OGA | 0 | DRD3 | 0.233429 |
| TUBB3 | 1 | THRA | 0 | HTR1B | 0.232092 |
| LGALS9 | 1 | PIM1 | 0 | ADRA2C | 0.230989 |
| NR1H2 | 1 | TYR | 0 | DHODH | 0.223757 |
| OGA | 1 | PTGER2 | 0 | CA12 | 0.218722 |
| PIM1 | 1 | PTGER3 | 0 | CES2 | 0.217547 |
| TYR | 1 | TBXAS1 | 0 | ADRA1A | 0.212227 |
| TBXAS1 | 1 | PTPRF | 0 | FABP3 | 0.210026 |
| RORA | 1 | RORA | 0 | CHRNA4 | 0.194868 |
