# Supplementary material for: Mechanisms of breast cancer treatment using Gentiana robusta: evidence from comprehensive bioinformatics investigation
Source: Sci Rep. 2024 Dec 30;14:31567. doi: 10.1038/s41598-024-76063-z (PMC11686125; doi:10.1038/s41598-024-76063-z)
Supplement: Supplementary file 11 — Supplementary Information 11. [file 41598_2024_76063_MOESM11_ESM.doc]

**Table S3.** XP and MM-GBSA results of EGFR

| Compound | Target | XP GScore | MM-GBSA dG Bind (kcal/mol) |
| --- | --- | --- | --- |
| QJ19 | EGFR | -9.041 | -24.33 |
| QJ1 | EGFR | -8.618 | -28.54 |
| QJ12 | EGFR | -8.292 | -21.3 |
| **QJ17** | EGFR | **-8.154** | **-30.51** |
| QJ22 | EGFR | -7.887 | -27.58 |
| QJ20 | EGFR | -7.823 | -18.42 |
| QJ5 | EGFR | -7.6 | 4.42 |
| QJ26 | EGFR | -7.478 | -45.66 |
| QJ10 | EGFR | -7.445 | -46.34 |
| QJ23 | EGFR | -7.281 | -35.12 |
| QJ18 | EGFR | -6.932 | -29.88 |
| QJ25 | EGFR | -6.924 | -40.41 |
| QJ9 | EGFR | -6.763 | -1.87 |
| QJ6 | EGFR | -6.592 | -13.66 |
| QJ16 | EGFR | -6.588 | -1.44 |
| QJ30 | EGFR | -6.414 | -40.07 |
| QJ3 | EGFR | -6.384 | -14.19 |
| QJ7 | EGFR | -6.325 | -17.76 |
| QJ13 | EGFR | -6.245 | -35.14 |
| QJ11 | EGFR | -6.071 | -33.57 |
| QJ21 | EGFR | -6.07 | -37.77 |
| QJ24 | EGFR | -6.07 | -37.75 |
| QJ2 | EGFR | -6.035 | -29.36 |
| QJ4 | EGFR | -5.6 | -27.8 |
| QJ32 | EGFR | -5 | -18.26 |
| QJ31 | EGFR | -4.677 | -33.59 |
| QJ39 | EGFR | -4.519 | -11.23 |
| QJ34 | EGFR | -4.288 | 6.2 |
| QJ33 | EGFR | -4.194 | -22.86 |
| QJ35 | EGFR | -4.078 | -36 |
| QJ36 | EGFR | -3.699 | -21.89 |
| QJ15 | EGFR | -3.668 | -18.73 |
| QJ29 | EGFR | -3.428 | -1.23 |
| QJ14 | EGFR | -3.366 | -14.89 |
| QJ37 | EGFR | -3.305 | -32.7 |
| QJ8 | EGFR | -3.08 | -8.19 |
| QJ27 | EGFR | -3 | -26.81 |
| QJ38 | EGFR | -2.598 | -0.55 |
| QJ28 | EGFR | -2.192 | -38.57 |
