# Supplementary material for: Mechanisms of breast cancer treatment using Gentiana robusta: evidence from comprehensive bioinformatics investigation
Source: Sci Rep. 2024 Dec 30;14:31567. doi: 10.1038/s41598-024-76063-z (PMC11686125; doi:10.1038/s41598-024-76063-z)
Supplement: Supplementary file 12 — Supplementary Information 12. [file 41598_2024_76063_MOESM12_ESM.doc]

**Table S4.** XP and MM-GBSA results of IL6

| **Compound** | **Target** | **XP GScore** | **MM-GBSA dG Bind (kcal/mol)** |
| --- | --- | --- | --- |
| **QJ25** | IL6 | **-8.528** | **-33.17** |
| QJ1 | IL6 | -8.343 | -26.79 |
| QJ22 | IL6 | -8.318 | -41.68 |
| QJ23 | IL6 | -7.587 | -27.89 |
| QJ10 | IL6 | -7.484 | -43.67 |
| QJ17 | IL6 | -6.885 | -43.66 |
| QJ2 | IL6 | -6.773 | -35.27 |
| QJ19 | IL6 | -6.765 | -33.86 |
| QJ20 | IL6 | -6.239 | -33.79 |
| QJ5 | IL6 | -6.175 | -22.66 |
| QJ3 | IL6 | -6.043 | -34.17 |
| QJ11 | IL6 | -5.934 | -17.13 |
| QJ8 | IL6 | -5.896 | -13.48 |
| QJ12 | IL6 | -5.879 | -30.26 |
| QJ7 | IL6 | -5.857 | -28.59 |
| QJ16 | IL6 | -5.778 | -26.05 |
| QJ18 | IL6 | -5.727 | -33.88 |
| QJ21 | IL6 | -5.718 | -32.05 |
| QJ13 | IL6 | -5.7 | -37.14 |
| QJ15 | IL6 | -5.685 | -29.3 |
| QJ9 | IL6 | -5.229 | -27.77 |
| QJ4 | IL6 | -5.182 | -26.02 |
| QJ26 | IL6 | -5.109 | -48.95 |
| QJ24 | IL6 | -4.94 | -32.78 |
| QJ32 | IL6 | -4.686 | -22.82 |
| QJ6 | IL6 | -4.652 | -33.74 |
| QJ31 | IL6 | -3.895 | -29.71 |
| QJ34 | IL6 | -3.795 | -12.05 |
| QJ30 | IL6 | -3.773 | -21.37 |
| QJ14 | IL6 | -3.734 | -29.3 |
| QJ39 | IL6 | -3.644 | -25.92 |
| QJ29 | IL6 | -3.633 | -15.67 |
| QJ36 | IL6 | -3.632 | -21.41 |
| QJ35 | IL6 | -2.989 | -23.82 |
| QJ33 | IL6 | -2.887 | -18.45 |
| QJ27 | IL6 | -2.883 | -30.67 |
| QJ37 | IL6 | -2.491 | -35.87 |
| QJ38 | IL6 | -1.87 | -15.06 |
| QJ28 | IL6 | 4.316 | -51.43 |
