# Supplementary material for: Mechanisms of breast cancer treatment using Gentiana robusta: evidence from comprehensive bioinformatics investigation
Source: Sci Rep. 2024 Dec 30;14:31567. doi: 10.1038/s41598-024-76063-z (PMC11686125; doi:10.1038/s41598-024-76063-z)
Supplement: Supplementary file 13 — Supplementary Information 13. [file 41598_2024_76063_MOESM13_ESM.doc]

**Table S5.** XP and MM-GBSA results of TP53

| **Compound** | **Target** | **XP GScore** | **MM-GBSA dG Bind (kcal/mol)** |
| --- | --- | --- | --- |
| QJ10 | TP53 | -8.753 | -29.42 |
| **QJ17** | TP53 | **-8.283** | **-31.32** |
| QJ19 | TP53 | -7.753 | -40.08 |
| QJ1 | TP53 | -7.501 | -12.38 |
| QJ20 | TP53 | -6.964 | -48.67 |
| QJ6 | TP53 | -6.791 | -49.59 |
| QJ13 | TP53 | -6.635 | -29.53 |
| QJ3 | TP53 | -6.572 | -22.85 |
| QJ26 | TP53 | -6.523 | -39.84 |
| QJ12 | TP53 | -6.478 | -37.58 |
| QJ11 | TP53 | -6.387 | -27.66 |
| QJ5 | TP53 | -6.316 | -17.98 |
| QJ2 | TP53 | -6.183 | -26.28 |
| QJ22 | TP53 | -6.034 | -51.15 |
| QJ8 | TP53 | -6.016 | -25.12 |
| QJ16 | TP53 | -5.899 | -30.54 |
| QJ4 | TP53 | -5.812 | -29.99 |
| QJ23 | TP53 | -5.795 | -28.73 |
| QJ18 | TP53 | -5.708 | -30.69 |
| QJ7 | TP53 | -5.571 | -30.5 |
| QJ15 | TP53 | -5.466 | -16.19 |
| QJ24 | TP53 | -5.256 | -17.75 |
| QJ9 | TP53 | -5.118 | -37.81 |
| QJ28 | TP53 | -5.036 | -35.12 |
| QJ14 | TP53 | -5.011 | -25.5 |
| QJ25 | TP53 | -4.673 | -32.12 |
| QJ30 | TP53 | -4.096 | -29.78 |
| QJ21 | TP53 | -3.452 | -29.17 |
| QJ32 | TP53 | -3.376 | -20.14 |
| QJ27 | TP53 | -3.366 | -27.36 |
| QJ29 | TP53 | -3.338 | -1.39 |
| QJ39 | TP53 | -2.945 | -30.48 |
| QJ31 | TP53 | -2.799 | -28.89 |
| QJ38 | TP53 | -2.724 | -21.56 |
| QJ37 | TP53 | -2.718 | -14.63 |
| QJ36 | TP53 | -2.405 | -26.19 |
| QJ35 | TP53 | -2.014 | -27.35 |
| QJ33 | TP53 | -1.943 | -19.64 |
| QJ34 | TP53 | -1.321 | -13.43 |
